# Unlocking the Potential of Push‐Pull Pyridinic Photobases: Aggregation‐Induced Excited‐State Proton Transfer

**DOI:** 10.1002/chem.202403388

**Published:** 2024-11-25

**Authors:** Letizia Mencaroni, Tommaso Bianconi, Maria Aurora Mancuso, Manju Sheokand, Fausto Elisei, Rajneesh Misra, Benedetta Carlotti

**Affiliations:** ^1^ Department of Chemistry Biology and Biotechnology and CEMIN University of Perugia 06123 Perugia Italy; ^2^ Department of Chemistry University of Wisconsin-Madison 53706 Madison USA; ^3^ Istituto di Tecnologie Avanzate per l'Energia ‘'Nicola Giordano'' (CNR-ITAE) 98126 Messina Italy; ^4^ Department of Chemistry Indian Institute of Technology 453552 Indore India

**Keywords:** Push-pull compounds, Aggregation-induced emission, Excited-state proton transfer, Photobases, Ultrafast spectroscopy

## Abstract

The pH effect on the photophysics of three push‐pull compounds bearing dimethoxytriphenylamine (TPA‐OMe) as electron donor and pyridine as electron acceptor, with different ortho‐functionalization (−H, −Br, and −TPA‐OMe), is assessed through steady‐state and time‐resolved spectroscopic techniques in DMSO/water mixed solutions and in water dispersions over a wide pH range. The enhanced intramolecular charge transfer upon protonation of the pyridinic ring leads to the acidochromic (from colorless to yellow) and acido(fluoro)chromic (from cyan to pink) behaviours of the investigated compounds. In dilute DMSO/buffer mixtures these molecules exhibited low pK_a_ values (≤3.5) and extremely short singlet lifetimes. Nevertheless, it is by exploiting the aggregation phenomenon in aqueous environment that the practical use of these compounds largely expands: i) the basicity increases (pKa≈4.5) approaching the optimum values for pH‐sensing in cancer cell recognition; ii) the fluorescence efficiencies are boosted due to Aggregation‐Induced Emission (AIE), making these compounds appealing as fluorescent probes; iii) longer singlet lifetimes enable Excited‐State Proton Transfer, paving the way for the application of these molecules as photobases (pK_a_*=9.1). The synergy of charge and proton transfers combined to the AIE behaviour in these pyridines allows tunable multi‐responsive optical properties providing valuable information for the design of new light‐emitting photobases.

## Graphical abstract

The pH‐dependent photophysics of three push‐pull pyridines is addressed through steady‐state and ultrafast spectroscopies. Aggregation is the key to multi‐responsive fluorophores: appealing pK_a_ values for cancer cell recognition; enhanced fluorescence efficiencies (φ_F_) for both neutral (**Py‐1**) and protonated (**Py‐1(H^+^)**) species, leading to acido(fluoro)chromism; longer singlet lifetimes (φ_F_) allowing the Excited‐State Proton Transfer (ESPT) crucial for applications as photobases.

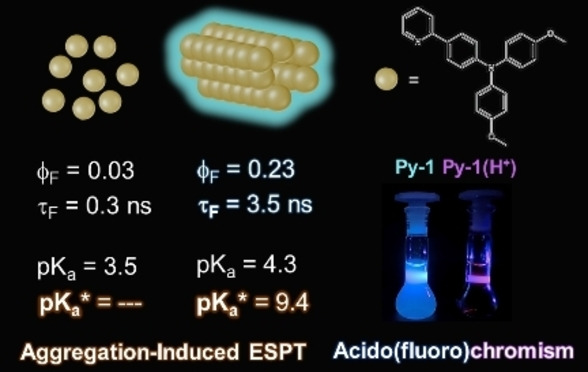

## Introduction

Conjugated organic molecules displaying a donor‐acceptor (D−A) type architecture have shown appealing properties to be exploited in a large variety of applications spanning from optoelectronics[^1^, ^2^] and photonics,[^3^, ^4^, ^5^] to photochemistry[^6^, ^7^] and biophysics allowing for both diagnosis, as fluorescent probes,[^8^, ^9^] and therapeutic action.[^10^, ^11^, ^12^, ^13^] Azo‐compounds such as pyridines have been found to be suitable building blocks for this kind of organic D‐π‐A molecules, namely push‐pull compounds, providing extreme tunability in their optical and photophysical properties thanks to the strong electron‐withdrawing ability and the ease of the chemical tailorability and functionalization.[^14^, ^15^, ^16^, ^17^, ^18^, ^19^, ^20^, ^21^] In addition, the inclusion of nitrogen‐heteroatoms in the D−A molecular structure opens the possibility to have protonable centers, hydrogen bond formation and chelation, therefore implying a multi‐stimulus responsive photobehaviour, expanding their applicability.^[22]^ By coupling the photoinduced intramolecular charge transfer (ICT) occurring from the electron‐donating to the electron‐deficient subunits of the push‐pull compounds and the proton transfer process involving the pyridinic nitrogen atom, one could in principle obtain fluorescent probes with pH‐sensitivity capable of real time and fast response, high sensitivity, and excellent selectivity. More recently, Aggregation‐Induced Emission (AIE) materials[^23^, ^24^, ^25^, ^26^, ^27^] have aroused deep interest as they promise to overcome the Aggregation‐Caused Quenching (ACQ) effect of traditional fluorophores and guarantee, at the same time, high sensitivity, processability, enhanced fluorescence also in biological environment.[^28^, ^29^, ^30^, ^31^, ^32^, ^33^, ^34^] Therefore pH‐sensitive fluorescent probes with AIE characteristic would be capturing materials to improve the performance of organic chemosensors and probes.[^35^, ^36^, ^37^, ^38^, ^39^] Triphenylamines derivatives have been identified as one of the best AIEgen candidates where the AIE, occurring via restricted intramolecular rotations of the aromatic rings in the aggregate state,[^40^, ^41^] showed to be coupled to a marked ICT character.[^42^, ^43^, ^44^] On the other hand, the coupling between AIE and excited state proton transfer (ESPT) has been rarely reported in the literature and mainly just in computational studies.[^45^, ^46^, ^47^, ^48^] ESPT has been sometimes described as the competitive process responsible for the solution monomer quenching of AIE‐active materials.^[49]^ This is where our present work wants to give a different and innovative contribution: using advanced time‐resolved spectroscopies to unfold the possible synergy between AIE and ESPT.

**Scheme 1:**
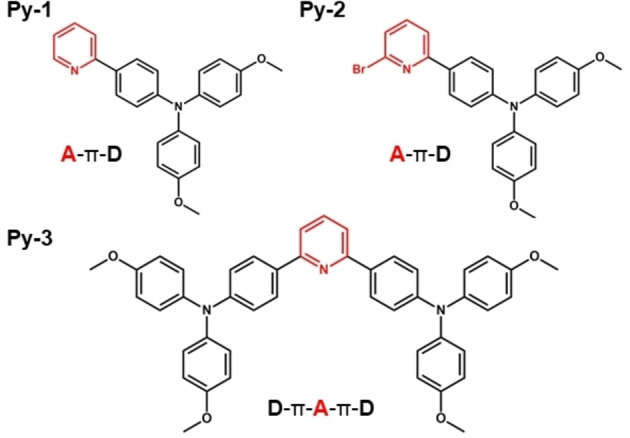
Chemical structures of the investigated *push‐pull* systems: the dimethoxytriphenylamine (TPA‐OMe) portion acts as the electron donor D (*push*, in black) while the heterocyclic pyridine as the electron acceptor (*pull*) and proton acceptor A (in red).

Recently, an intriguing class of organic push‐pull dipolar and quadrupolar derivatives, bearing dimethoxytriphenylamine (TPA‐OMe) as electron donor (D) and pyridine as electron acceptor (A) differently functionalized at the *ortho* position (−H, **Py‐1**, −Br, **Py‐2**, and −TPA‐OMe, **Py‐3**, Scheme 1), was synthetized and fully characterized for the structure‐property relationships in many solvents of different polarities.^[14]^ The photoinduced ICT, triggered by the different D/A substituents in the molecular architectures, was found to play a key role in determining the environment‐ and stimuli‐responsive optical properties of the investigated fluorophores.^[14]^ Interestingly, these materials not only resulted to be highly fluorescent in solution and, particularly, in the solid state, thanks to the AIE phenomenon, but also showed mechanochromic luminescence and considerable Non‐Linear Optical (NLO) properties. Herein, an in‐depth analysis of the influence of pH on the spectral and photophysical properties and on the excited‐state evolution of these push‐pull pyridine derivatives was carried out through the application of steady‐state and ultrafast spectroscopies. Particularly, time‐resolved measurements proved essential to explore the multitasking nature of the pyridinic ring, acting both as electron and proton acceptor, unveiling the possible mutual interplay between ESPT and ICT. Firstly, spectrophotometric and fluorimetric titrations were performed in DMSO/water (60/40 %v/v) mixtures to gain insight into the acid/base behavior of the investigated compounds. Later on, the study of the pH‐dependence of the photophysics of these fluorophores was also extended to their nanoaggregates in DMF/water (1/99 %v/v) dispersions, with the aim of combining the AIE behavior with the environment‐responsive properties.

## Results and Discussion

### Acid‐Base Properties in Solution

**Figure 1:**
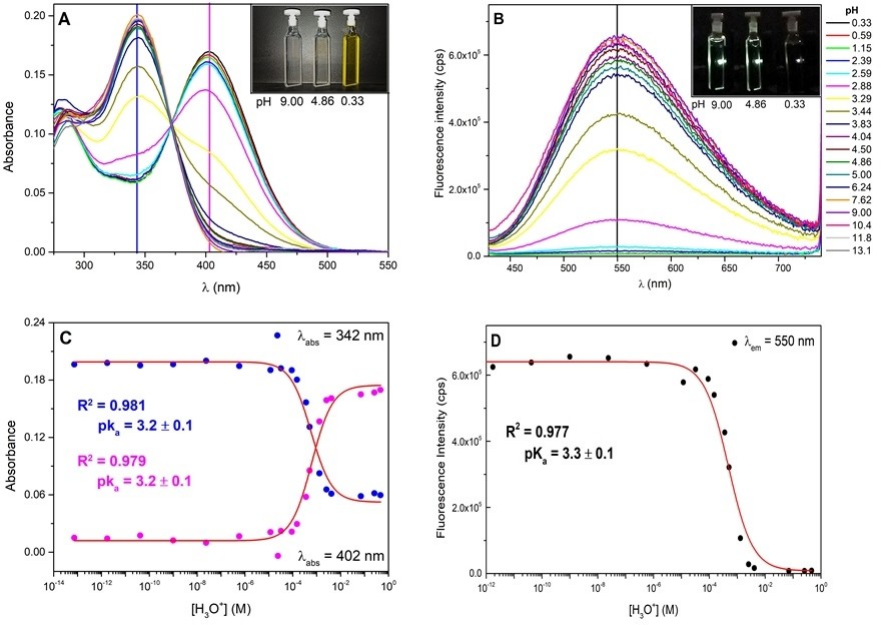
Spectrophotometric (A) and fluorimetric (B) titrations of compound **Py‐1** in a large pH range (DMSO/buffer 60/40 %v/v). Fluorescence spectra were obtained by exciting each sample at the isosbestic point (λ_exc_=372 nm). Insets: (A) from left to right **Py‐1** at pH=9, 4.86, 0.33; (B) from left to right **Py‐1** at pH=9, 4.86, 0.33 under blue laser irradiation. pK_a_ fittings obtained from the spectrophotometric (C) and fluorimetric (D) titrations for compound **Py‐1** in DMSO/buffer 60/40 (%v/v).

The impact of the medium acidity on the steady‐state absorption and emission spectra of the three push‐pull pyridine derivatives under investigation was firstly addressed in DMSO/buffer mixtures (retaining 60 %v/v of DMSO, Figure S1) due to the reduced solubility of the sample in pure water. Under these experimental conditions, the pH changes on the photobehaviour of the well‐dispersed monomers could be probed. In fact, in a previous study aggregated species proved to form when the water amount in such mixtures exceeded 80 %.^[14]^ The main results of the pH‐dependent spectral properties of the three compounds are collected in Figure 1 for **Py‐1**, selected as the representative example, and in Figures S2 and S3 for **Py‐2** and **Py‐3**, respectively. As depicted in Figure 1, the absorption spectrum initially peaked at 342 nm in DMSO/buffer mixture at neutral or basic pHs progressively disappears upon increasing the acidity of the environment in concomitance with the appearance of the bathochromic band centred at 402 nm. As already observed for other pyridine‐containing compounds,[^22^, ^50^, ^51^, ^52^, ^53^] the red shift of the absorption spectrum is in line with the protonation of the pyridinic nitrogen atom. As a matter of fact, the protonation of the pyridine would be responsible for an increased electron‐withdrawing strength of the acceptor in these push‐pull molecules. Therefore, an enhanced intramolecular charge transfer character is expected to push the S_0_–S_1_ electronic transition of the protonated sample, **Py‐1(H^+^)**, towards longer wavelengths. As for the cases of **Py‐2** and **Py‐3** (Figures S2 and S3), a similar reddening of the absorption spectra, by ca. 60 and 80 nm respectively, was observed upon increasing the [H_3_O^+^] concentration. As a result, all the three substrates showed a mild acidochromic behaviour, with the colourless solution turning yellow upon protonation (Figure 1A, inset). For all the investigated molecules an isosbestic point located at 372 nm for **Py‐1**, at 384 nm for **Py‐2** and at 396 nm for **Py‐3** evidenced the presence of two species in chemical equilibrium over the wide investigated pH range. In fact, even though the second protonation of the triphenylamine portion would be in principle possible, it was never observed as it would require very acidic environments (pK_a_<−4, see below). The isosbestic points were then chosen as excitation wavelengths for the fluorimetric analysis. Generally, the fluorimetric titrations pointed out the presence of a bright emissive spectrum peaked in the green region of the visible for the neutral fluorophore (550 nm for **Py‐1**, 578 nm for **Py‐2** and 527 nm for **Py‐3**), slightly red‐shifted if compared to the pure DMSO solution (λ_em_~520 nm)^[14]^ due to the more polar environment in the DMSO/buffer mixture. More importantly, the fluorescence is gradually reduced upon increasing the medium acidity. As for compound **Py‐1** shown in Figure 1B, the fluorescence spectrum appeared to be completely quenched at pH values <2.5, while higher [H_3_O^+^] concentrations were required to further dampen the emission signal in the cases of **Py‐3** and, particularly, **Py‐2** (Figures S2 and S3). The spectrophotometric titrations were then monitored at suitable wavelengths, corresponding to the absorption maxima featured by the neutral and protonated pyridine species (see the blue and magenta vertical lines in Figure 1A referring to the **Py‐1** and **Py‐1(H^+^)** absorption, respectively), to determine the pK_a_ value for this acid/base equilibrium at the ground state level. From the fitted sigmoid functions depicted in Figure 1C, a pK_a_=3.2 value was calculated for **Py‐1** in DMSO/buffer mixture, which resulted to be higher than those provided by the same treatments for **Py‐2** (pK_a_~0.6 Figure S4A) and **Py‐3** (pK_a_=3.1, Figure S5A). To be fair, in the case of the brominated‐derivative the plateau associated to the protonated species (**Py‐2(H^+^)**) could not be reached, even when using HClO_4_ to lower the pH of the DMSO/water mixture down to H_0_=−5 (Figure S2). The more accurate pK_a_ values provided by the global fitting procedure of the multivariate spectroscopic data through ReactLab Equilibria software (Figures S5–S8) were then collected in Table 1. The different acidity of the compounds (Table 1) could be easily rationalized by considering the diverse electron donating/accepting strength inferred by the lateral *ortho*‐functionalization of the three pyridines derivatives. As a matter of fact, the difference between the dipolar **Py‐1** (pK_a_~3.2) and the quadrupolar **Py‐3** (pK_a_~3.0) compounds may lie in the relatively greater steric hindrance on the same pyridine unit in the double‐armed derivative. On the contrary, the inductive effect exerted by the bromine atom causes a depletion of the electronic charge density on the pyridine itself, thus making the protonation more difficult to be achieved, yielding a much lower pK_a_. Particularly, the pK_a_ values obtained for the investigated series were found to perfectly align that reported in the literature for the parent Pyridine‐H^+^ compound in DMSO (pK_a_=3.45).^[54]^ Unfortunately, the fluorimetric titrations could not provide any information on the equilibrium rate constant for the first singlet excited state (pK_a_*) as concluded by the absence of any additional inflection on the sigmoidal curves in Figures 1D and S4B–S5B. Therefore, the Förster‐Weller cycle model was employed to obtain a tentative esteem, reported in Table 1, for the pK_a_* values of the pyridine derivatives under study.

**Table 1:** Acid‐base properties of the investigated compounds as derived by spectrophotometric (pK_a,abs_) and fluorimetric (pK_a,em_) titrations in DMSO/buffer mixture (60/40 %v/v) analyzed by global fitting procedure through ReactLab Equilibria software. Last column shows the pK_a_* values calculated by using the Förster cycle (pK_a_*_th_) with data collected in Figure S6.

Compound	λ_abs_/nm	pK_a,abs_	λ_em_/nm	pK_a,em_	pK_a_*_th_
**Py‐1**	342	3.212±0.004	550	3.302±0.004	11.2
**Py‐1(H^+^)**	402		625		
**Py‐2**	360	0.02±0.01	578	0.61±0.01	7.2
**Py‐2(H^+^)**	420		585		
**Py‐3**	360	3.07±0.01	527	2.88±0.01	11.6
**Py‐3(H^+^)**	443		625		

### Quantum‐Mechanical Calculations

**Figure 2:**
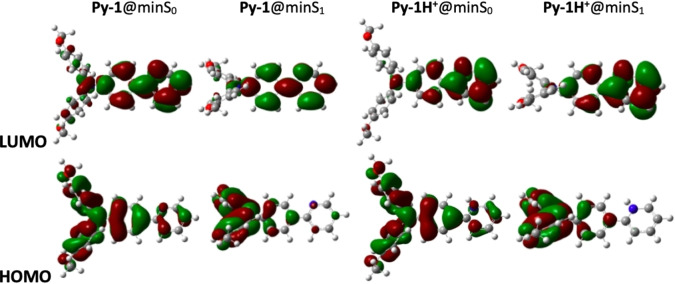
Frontier molecular orbitals for neutral and protonated **Py‐1** at the ground (min S_0_) and excited state (min S_1_) optimized geometry.

A comprehensive computational investigation was performed for both the neutral and the protonated forms of the investigated molecules via DFT and TD‐DFT. All the obtained results are shown in detail in Section S7 of the Supporting Information. The computed optimized geometries exhibited a large structural rearrangement when passing from the ground to the excited state, with the two methoxy‐phenyl rings of the triphenylamine donor unit becoming closer to each other resulting in a more twisted geometry (Figures 2 and S20). A similar, even though less apparent, structural change was observed upon protonation of the pyridinic nitrogen, when passing from the neutral to the protonated compounds. The computational prediction of the experimental absorption and emission spectra was found to be remarkable in all cases (see Tables S5–S10 and Figures S24, S32 and S38). Generally, both the low energetic S_0_→S_1_ absorption and the S_1_→S_0_ emission transitions were found to be mainly described by the HOMO‐LUMO configuration (see Figure 2 for the case of **Py‐1** and the Supporting Information for complete results), as shown by the Natural Transition Orbital (NTO) analysis in Table S11. The HOMO is mainly localized on the triphenylamine donor and the LUMO on the pyridine acceptor. It is noteworthy that the charge separation significantly increases in the excited state relative to the ground state and in the protonated relative to the neutral molecule (Figure 2). The oscillator strength for the S_0_→S_1_ absorption was found to be large (above 1), while being reduced for the S_1_→S_0_ emission, particularly for the protonated species. This finding is in agreement with the pH effect on the experimental fluorescence quantum yields and rate constants (see Figure 1B and Table 2 below). The frontier molecular orbitals obtained for the neutral **Py‐3** show symmetrical charge displacement from the lateral donors to the central pyridine acceptor during the absorption transition while suggest the occurrence of symmetry breaking charge transfer in the excited state (Figures S35 and S36). The excited state optimization for the protonated forms was challenging and could be successfully completed in the case of the simpler **Py‐1(H^+^)** compound. In fact, while the minimum of the potential energy surface of the ground state was unambiguously reached for a 20° dihedral angle between the methoxy‐diphenylamine and the central phenyl, many more accessible angles were possible in the excited state at room temperature (Figure S28). The excited state absolute minimum was found to be in correspondence of a 60° dihedral angle, suggesting a significantly twisted excited state geometry for the protonated **Py‐1(H^+^)**, consistently with the largely red shifted emission at ca. 690 nm. The twisted excited state geometry indeed favours the enhanced charge separation for the protonated form of these push‐pull molecules.

**Table 2:** Fluorescence quantum yields (φ_F_), fluorescence lifetimes (τ_F_) and derived radiative kinetic rate constants (k_F_=φ_F_/τ_F_) measured for the investigated compounds in DMSO/buffer mixtures (60/40 %v/v) at different pHs.

pH	**Py‐1**			**Py‐2**			**Py‐3**		
	φ_F_	τ_F_/ps^[b]^	k_F_/10^8^ s^−1^	φ_F_	τ_F_/ps^[b]^	k_F_/10^8^ s^−1^	φ_F_	τ_F_/ps	k_F_/10^8^ s^−1^
9.00^[a]^	0.03	270	1.1	0.03	360	0.83	0.20	τ_F,1_=200 (7 %)^[c]^ τ_F,2_=2400 (93 %)^[c]^	0.83
4.86	0.03	380	0.78	0.02	460	4.4	0.20	τ_F,1_=200 (7 %)^[c]^ τ_F,2_=2300 (93 %)^[c]^	0.85
<1	0.0004	22	0.18	0.001	27	3.7	0.0004	44^[b]^	0.091

^[a]^ pH=11.8 in the case of **Py‐3**. ^[b]^ from fs‐FUC data (see Table S2). ^[c]^ from ns‐TC‐SPC results (λ_exc_=377 nm).

### Proton‐Enhanced Ultrafast Intramolecular Charge Transfer Dynamics in Solution

All the pH‐induced changes on the fluorescence properties, namely fluorescence quantum yields, lifetimes and relative kinetic rate constants, of the three investigated molecules were summarized in Table 2. Interestingly, **Py‐3** retained a great emissive capability in these aqueous solutions, at least in basic environment (φ_F_~0.20), if compared to the **Py‐1** and **Py‐2** substrates (φ_F_=0.03 at pH=9), for which the dipolar character of the structure likely favours the ICT rather than the radiative process.

As inferred by the fluorescence quenching underlined by the fluorimetric titrations, the emitting capability of the pyridine substrates dropped down by several orders of magnitude upon moving from pH~11 down to pH<1 (Tables 2 and S1). The largest reduction of the fluorescence quantum yield was detected in the case of the quadrupolar **Py‐3** (φ_F_=0.20 at pH=11.8 and 0.0004 at pH<1). In line with the spectral modifications discussed above, the fluorescence quenching could also be explained by considering an enhanced intramolecular charge transfer character upon protonation of the pyridine unit, which would lead to the preferential non‐radiative deactivation of the polarized S_1_ state. The lowering of the fluorescence quantum yields was paralleled by the decrease of the radiative lifetimes when shifting the acid‐base equilibrium towards the monoprotonated species, that is when pH<pK_a_. In this regard, the fluorescence lifetime measurements were firstly addressed via ns TC‐SPC technique and, later on, through broadband Fluorescence UpConversion (fs‐FUC) with improved temporal resolution (Table S2).

In this regard, the combined application of fs‐FUC and Transient Absorption (fs‐TA) experiments also allowed for a deeper insight into the excited state evolution of the investigated molecules as a function of pH, revealing a peculiar “interplay” between the intramolecular charge transfer and proton transfer processes.

**Figure 3:**
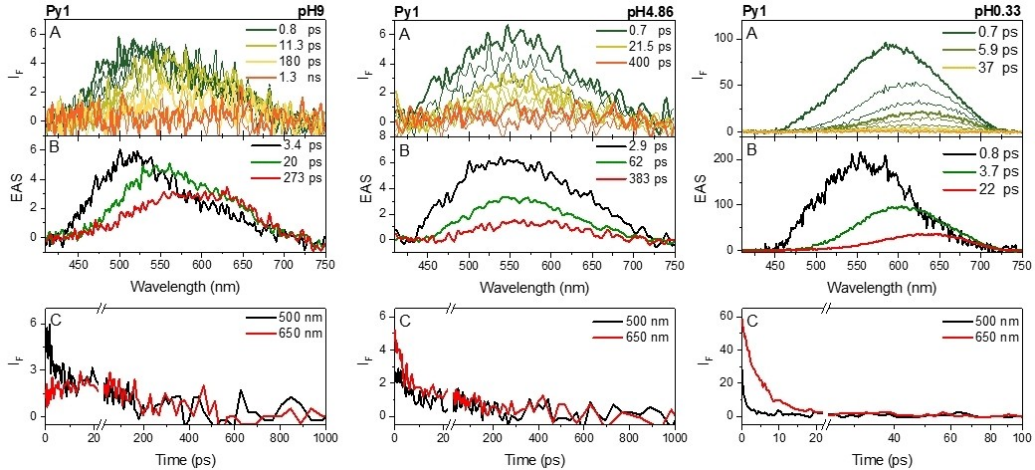
Fs‐FUC (λ_exc_=400 nm) results for **Py‐1** in DMSO/buffer 60/40 (%v/v) at different pHs. Panel A: spectra obtained at different delay times after excitation. Thicker lines refer to representative spectra corresponding to the explicit time delays (see legend). Panel B: Evolution Associated Spectra (EAS) obtained for the transients provided by Global Analysis with related lifetimes: Solv./S_1,LE_ (solvation/locally‐excited singlet state, black line), Solv./VC (solvation/vibrational cooling, brown), SR (structural relaxation, green line), and S_1,ICT_, (stabilized singlet state with ICT nature, red line). Panel C: Representative kinetics acquired at selected wavelengths to probe the S_1,LE_, black, and S_1,ICT_, red, deactivation dynamics.

The fs‐FUC results obtained at different pHs (pH=9, 4.86 and 0.33) were collected in Figure 3 for **Py‐1**, as representative example. The ultrafast dynamic of the solutions with a pH>pK_a_, namely pH=9 and 4.86, presented very similar spectral evolution and transient lifetimes: at early time delays, the fluorescence spectrum appears as a broad band centred at ca. 520 nm at pH=9 or 550 nm at pH=4.86 (see the olive spectra depicted in panels A of Figure 3) which then progressively red shifts, while decaying, to reach the bathochromic emission profile centred at ca. 590 nm (red curves in Figure 3). The increased acidity of the medium when moving from pH=9 to 4.86, upon approaching the pK_a_ value, was responsible for the stabilization of the initial position of the olive spectrum due to the enhanced charge transfer character of the structure. At pH<pK_a_ (pH=0.33, Figure 3, right panel) the fluorescence is initially located further in the red, at ca. 575 nm, and then rapidly evolves to give the broad emission at ca. 625 nm in few tens of picoseconds. The complementary fs‐TA experiments (Figure S7) basically confirmed the same acido(fluoro)chromic behaviour of the **Py‐1**/**Py‐1(H^+^)** fluorescence as highlighted by the red shift of the negative signals (▵A<0) corresponding to the stimulated emission process when lowering the pH. Unfortunately, the fs‐TA could not provide additional information relative to the fs‐FUC as the Excited State Absorption (ESA) bands were only partially visualized at the lateral edges of our spectral window. However, the Global Analysis of the fs‐FUC and fs‐TA data disclosed the presence of three transient species describing the overall excited‐state pH‐dependent photodynamics for **Py‐1**. When the neutral pyridine prevailed (pH=9 and 4.86), the excited‐state deactivation dynamic presented: i) the first transient, with τ_1_~3 ps (see the black line in panels C of Figures 2 and S7), associated to the locally excited (LE) singlet state, concomitant to the solvation (Solv.) induced by the polar medium; ii) a green transient featured by a lifetime of tens of picoseconds assigned to some structural relaxation (SR) process; iii) the strongly stabilized singlet state having an ICT nature, shown in red, characterized by a lifetime of ca. 300 ps. At pH<pK_a_ (Figures 2, S7 and Table S2), where the excited state deactivation is abruptly accelerated, the first S_1,LE_ state of **Py‐1(H^+^)** decays in less than 1 ps to give, after a rapid vibrational cooling (VC) aided by the solvent reorganization, the short‐lived S_1,ICT_ (τ_F_=22 ps) of the protonated species, peaked at 630 nm. As for the remaining compounds, **Py‐2** and **Py‐3**, the excited‐state evolution did not substantially differ from that of **Py‐1** (Table S2 and Figures S8–S11). Unfortunately, the fs‐FUC measurements in the case of compound **Py‐3** in basic environment were prevented by solubility issues. As expected from our previous work on the solvent effect,^[14]^ the increased polarity of the DMSO/buffer mixture caused the first drop of the singlet lifetimes from the nanosecond time‐scale in the pure DMSO solutions (τ_F_=5.6 ns for **Py‐1** and τ_F_=0.95 ns for **Py‐2**)^[14]^ down to few hundreds of picoseconds for the dipolar **Py‐1** (τ_F_=273 ps at pH=9) and **Py‐2** (τ_F_=365 ps at pH=9) compounds. When it came to the protonated forms, a further decrease of one order of magnitude was observed (τ_F_=22 ps for **Py‐1(H^+^)** and τ_F_=27 ps for **Py‐2(H^+^)**). This result could be easily rationalized considering that the ICT nature of the emitting singlet excited state would be even more emphasized by the strongest electron‐withdrawing behaviour of the pyridine in its acid form. In this sense, the protonation proved to boost the ultrafast intramolecular charge transfer. Longer fluorescence lifetimes for the relaxed S_1_ state were observed for the neutral **Py‐3** (few nanoseconds) relative to **Py‐1** and **Py‐2** (hundreds of picoseconds). Surprisingly, the most pronounced lifetime quenching upon decreasing pH was observed for the quadrupolar **Py‐3** compound, in line with the trend of the fluorescence efficiency. In fact, the τ_F_ was found to vary from 2.4 ns at neutral and basic pH, typical of a Planar ICT (PICT) state (τ_F_=5.6 ns in pure DMSO),^[14]^ down to tens of picoseconds (τ_F_=44 ps), assignable to a Twisted ICT (TICT) state (Table S2).

As a matter of fact, a closer look onto the fs‐FUC results unveiled the close resemblance of **Py‐1** and **Py‐3** and, particularly, the almost perfect overlap of the **Py‐1(H^+^)** and **Py‐3(H^+^)** emission spectra (Figure S12). The symmetry breaking on the S_1,ICT_ state^[55]^ might be called into question, which would also explain all the similarities with the dipolar analogue in the fluorescence spectrum, quantum yield and lifetime in the case of the protonated species. Therefore, one can speculate that the protonation might not only assist the intramolecular charge transfer but also enable the symmetry breaking in the case of **Py‐3**. Additionally, the extremely short singlet lifetimes (Table 2) might be the reason why the acid‐base re‐equilibration in the excited state, although predicted by the Förster‐Weller cycle, was not detected by fluorimetric analyses, as it failed to compete with the ultrafast deactivation of S_1_ via radiative and non‐radiative processes.[^22^, ^56^, ^57^]

Under these experimental conditions (dilute DMSO/buffer 60/40 %v/v mixtures) the investigated pyridines did not prove to be attractive in terms of applications if one considers the rather low pK_a_ value, which limits their applicability as environment‐sensitive probes. Nevertheless, these push‐pull compounds manifested an alluring AIE phenomenon in DMF/water mixtures which implied, as a start, an increased fluorescence efficiency in aqueous suspensions and then, an exalted color tunability, i. e. mechanochromic luminescence.^[14]^ Aiming at exploiting the enhanced sensitivity of the spectral and photophysical properties, the pH effect was thus carefully probed on the aggregated species of the compounds under study.

### Acid‐Base Properties of Aggregated Species

**Figure 4:**
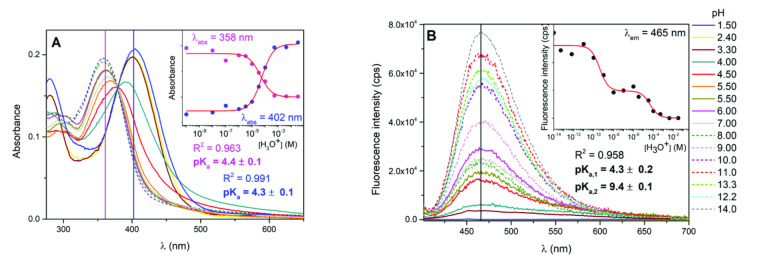
Spectrophotometric (A) and fluorimetric (B) titrations together with relative pK_a_ fittings (insets) obtained for compound **Py‐1** in water dispersion [DMF/buffer (1/99 %v/v)] in a large pH range. Fluorescence spectra were obtained by exciting each sample at the isosbestic point (λ_exc_=379 nm).

The impact of the medium acidity on the spectral properties of **Py‐1** aggregates obtained in water dispersion (DMF/buffer 1/99 %v/v) was reported in Figure 4. Information about the size and packing of these nanoaggregates was obtained in a previous work^[14]^: aggregates characterized by diameters of hundreds of nanometers were formed. Similar to what previously discussed for the monomers in dilute solutions, the addition of [H_3_O^+^] to the environment led to a red shift of the absorption spectrum of **Py‐1**, initially centered at 358 nm for the neutral form, towards the **Py‐1(H^+^)** peak located at 402 nm (Figure 4A). The best fittings of the absorbance changes as a function of pH in water dispersion, shown in Figure 4A (inset), yielded a pK_a_ value of about 4.3 for **Py‐1** to be compared to pK_a_=3.21 in the case of the DMSO/buffer mixed solution. The increased acid‐base equilibrium constant of almost one order of magnitude could be explained by the aggregation phenomenon together with the absence of DMSO (acting as a strong base, thus lowering the pH and the derived pK_a_).^[58]^ Notably, the experimentally derived pK_a_ value (≈4.5) for compound **Py‐1** resulted to be particularly useful for following the intracellular pH variations in biological fluids and living cells, i. e. for the more acidic lysosomal lumen of cancer cells (pH 4.5 and 5.5 vs 5.0 or 6.0 in normal cells).[^59^, ^60^, ^61^] As a result, **Py‐1** becomes promising as potential diagnostic agent. Moreover, the experimental pK_a_=4.3 was found to closely approach the value simulated by the ACD/Labs analysis, predicted to be pK_a,calc_=4.96 for the pyridinic nitrogen protonation in pure water (Table 3). More importantly, a fluorimetric titration was carried out for **Py‐1** at different pHs in DMF/water suspension [DMF/buffer (1/99 %v/v)]. The spectral changes as a function of pH of the **Py‐1** fluorescence band, peaked at 465 nm at pH>4, are shown in Figure 4B unveiling its acido(fluoro)chromic behavior. As a matter of fact, the fluorescence signal of the neutral species is progressively toned down upon increasing the acidity of the medium, while a weak emissive profile, centered at 650 nm, is observed for pH<pK_a_, assigned to **Py‐1(H^+^)**. The latter finding demonstrates that the cyan fluorescence of **Py‐1** could be turned pink by increasing the [H_3_O^+^] concentration, that is when **Py‐1(H^+^)** is excited (Figure S13). The reddening of both absorption and emission spectra of the protonated pyridinium and above all the huge Stokes Shift measured in the case of **Py‐1(H^+^)** (▵ν=9500 cm^−1^) were again rationalized considering the enhanced charge transfer character of the excited singlet state upon protonation.

**Table 3:** Acid‐base properties of **Py‐1** aggregates in water dispersion derived by spectrophotometric, pK_a,abs_, and fluorimetric, pK_a,em_, titrations analyzed by best fitting procedures through ReactLab Equilibria software and predicted by ACD/Labs software (pK_a,calc_). Last column shows the pK_a_* values calculated by using the Förster cycle (pK_a_*_th_).

**λ_abs_/nm**	**pK_a,abs_**	**λ_em_/nm**	**pK_a,em_**	pK_a_*_calc_	pK_a_*	pK_a_*_th_
358	4.27±0.01	465	4.25±0.02	4.96; −4.57^[a]^	9.14±0.02	12.4
402		640				

^[a]^ The second pK_a,calc_ value refers to the possible protonation of the triphenylamine unit.

The fitting at the representative emission wavelength (Figure 4B) surprisingly showed the presence of two acid‐base equilibria. The first pK_a,1_=4.3 perfectly reproduced that obtained for the analogous treatment of the spectrophotometric data (Figure 4A) which was thus assigned to the ground state equilibrium. More importantly, a second inflection yielding pK_a,2_=9.4 was highlighted. The global fitting procedure applied on these spectroscopic data, reported in Table 3, provided more accurate pK_a_ values: pK_a,1_=4.25 and pK_a,2_=9.14 accounting for two different equilibrium constants when the first singlet excited state gets involved. This finding would suggest the possible re‐equilibration phenomenon during the singlet excited state lifetime, that is an Excited‐State Proton Transfer (ESPT) before the S_1_ relaxation. When probing the same pH effect onto the aggregated species of **Py‐2** and **Py‐3** compounds, the study of the effect of medium acidity showed unexpected outcomes. As a matter of fact, while the appearance of a bathochromic absorption band could be followed at ca. 400 nm pointing to the protonation of the pyridinic nitrogen (Figure S13), it was not possible to detect a precise isosbestic point neither in the case of the brominated‐ nor the quadrupolar derivative. Furthermore, when lowering the pH below 0, additional absorption bands aroused at the red edge of the spectral window. We reckon that the stability of these nanoaggregates was compromised by the environment acidity and, therefore, some degradation might be responsible for the observed experimental outcomes. Also, as obtained for **Py‐2** in DMSO/water solution, it was not possible to fully shift the acid‐base equilibrium towards the protonated species **Py‐2(H^+^)** even at extremely acidic conditions (Figure S13). For this reason, a rigorous treatment to obtain the pK_a_ values was not performed for **Py‐2** and **Py‐3**. However, the simulation of the pK_a_ gained through the ACD/Labs software (Table S3) suggested the same trend of the pK_a_ values already found for the monomers in DMSO/buffer mixtures: **Py‐1**>**Py‐3**≫**Py‐2**. Additionally, the Förster‐Weller cycle came into aid to estimate the pK_a_* value for **Py‐1** and for **Py‐3** (Figure S14) in the absence of fluorimetric titrations, from the intersections of the emission and absorption spectra of the neutral and protonated species of the aggregates in water (Tables 3 and S3).

### Aggregation‐Induced Excited‐State Proton Transfer

To gain insight into the effect of pH on the fluorescence properties of the investigated aggregates, three representative water dispersions at different pHs (11, 7 and 1) were fully characterized (Table S4). As expected, the fluorescence capability of the **Py‐1** and **Py‐2** derivatives (φ_F_≥0.20) was largely enhanced in the aggregated forms if compared to the DMSO/water solutions (φ_F_~0.03) at pH>pK_a_ thanks to the marked AIE behaviour.^[14]^ As for **Py‐3**, instead, for which a negligible AIE effect was previously observed,^[14]^ an almost halved φ_F_ value (φ_F_=0.11, Table S4) was measured upon aggregation in basic environment (φ_F_=0.20 for the monomeric form in DMSO/buffer mixture, Table 2).

When the pH was reduced below the pK_a_, an evident fluorescence quenching of the molecules under study was found due to the protonation of the pyridine unit, implying a more efficient ICT at the expense of the radiative deactivation channel (i. e. φ_F_=0.23 at pH=11 and φ_F_=0.001 at pH=1 for **Py‐1**, Table 4). Even though upon protonation the emission efficiency was still drastically damped, the fluorescence quantum yields showed improved values of ca. one order of magnitude relative to those calculated for the protonated pyridines in solution (φ_F_=0.0004 for **Py‐1**, Table 2). A thorough photophysical characterization of the emission properties of the aggregates was carried out in the case of **Py‐1** to better confirm the possible re‐equilibration in the singlet excited state. In fact, as illustrated in Table 4, the φ_F_ values were found to gradually decrease as the equilibrium was shifted towards **Py‐1(H^+^)**. As a matter of fact, at intermediate pH values (pK_a_<pH<pK_a_*), i. e. pH=5 or 6, being the neutral **Py‐1** the expected prevailing species in the ground state (Figure S16D), the fluorescence quantum yield should show values of ca. 20 %, constant throughout the entire pH>pK_a_ range. Instead, φ_F_ values equal to 0.05 and 0.06 as for pH=5 and 6, respectively, were measured. Therefore, one can speculate that the conjugated acid and base forms both contribute to the radiative decay even when pH>pK_a_. This finding would be in line with the presence of an additional equilibrium (driven by pK_a_*) in the excited state of **Py‐1** aggregates upon photoexcitation, as evidenced by the relative fluorimetric titration (Figure 4B). These results would imply that even when exciting the conjugated base, the protonated **Py‐1(H^+^)** would still be present in the excited state, at least partially, affecting the fluorescence efficiency. The fitting of the fluorescence kinetics of the pyridine aggregates in water dispersion revealed the third‐exponential nature of the radiative decays, as expected by previous observation.^[14]^ The obtained lifetimes were reported in Table S4 for three representative pH values. These fluorescence decay times were found to be at least one order of magnitude longer than those measured in the dilute monomer solutions (Table 2) due to the AIE effect,[^62^, ^63^, ^64^] even when pH>pK_a_. Interestingly, in accordance with the fluorescence efficiency, the singlet lifetimes of the aggregated **Py‐1** sample gradually decreased upon lowering the pH (Table 4). It can be noted that two long‐lived fluorescent components of about 1 and 3.5 ns in basic environment were progressively shortened to finally give τ_1_=160 ps and τ_2_=650 ps in the case of the pure **Py‐1(H^+^)** species at pH=1. Surprisingly, at pH=6 or 7 (more generally in the pH range between pK_a_ and pK_a_*) two averaged values of fluorescence lifetimes (ca. 800 ps and 2.5 ns) were measured, confirming the presence of partial re‐equilibration at the S_1_ level. A rough estimation of the average radiative rate constant was also reported in Table 4 (last column) indicating a fully‐allowed emission transition at pH=11, being the k_F,av_≈1×10^8^ s^−1^, and an almost forbidden transition in the case of the pure protonated **Py‐1(H^+^)** at pH=1, k_F,av_≈3×10^6^ s^−1^, where the ICT in fact outcompetes the radiative channel. In absence of any re‐equilibration in the excited state, a constant fluorescence quantum yield, lifetime and kinetic rate constant should be observed until pH<pK_a_.^[22]^ However, the k_F,av_ kinetic parameter proved to be progressively reduced when increasing [H_3_O^+^] concentration. This evidence might be interpreted as another body of proof for the coexistence of both conjugated forms **Py‐1** and **Py‐1(H^+^)**, in the excited state in such intermediate neutral and mild acidic environments, due to the occurrence of ESPT. The most considerable drop on k_F,av_ was then observed between pH=5 and 4, where the acid‐base equilibrium at the ground state level is totally shifted towards the conjugated acid.

**Table 4:** Photophysical properties of **Py‐1** aggregates in water dispersion [DMF/buffer (1/99 %v/v)] at different pHs. Fluorescence lifetimes were measured through ns TC‐SPC (λ_exc_=377 nm). The kinetic rate constant reported in the last column (k_F,av_) was estimated by dividing the relative φ_F_ value for the averaged number obtained from the two longer fluorescence lifetimes. k_F,av_ obtained as: φ_F_/τ_F,av_ with τ_F,av_ being the weighted average lifetimes considering the two longer‐lived contributions.

pH	λ_abs_/nm	λ_em_/nm	φ_F_	τ_F_/ns	k_F,av_*/10^8^ s^−1^
11	358	466	0.23	0.20 (19 %) 1.1 (46 %) 3.5 (35 %)	1.00
10	358	465	0.15	0.20 (19 %) 1.1 (47 %) 3.6 (34 %)	0.63
9	358	465	0.14	0.20 (23 %) 1.1 (46 %) 3.4 (31 %)	0.62
8	362	465	0.10	0.20 (30 %) 0.90 (45 %) 3.0 (25 %)	0.50
7	365	465	0.07	0.20 (34 %) 0.80 (41 %) 2.5 (26 %)	0.43
6	362	465	0.08	0.20 (33 %) 0.80 (43 %) 2.9 (24 %)	0.43
5	378	465	0.05	0.20 (46 %) 0.60 (33 %) 2.0 (19 %)	0.38
4	392	465	0.02	0.20 (32 %) 1.1 (45 %) 4.1 (24 %)	0.077
1	402	650	0.001	0.16 (53 %) 0.65 (47 %)	0.025

The combined effect of the restricted microenvironment provided by aggregation, which proved to lengthen the fluorescence lifetimes by at least one order of magnitude, jointly to the abundant protic solvent (H_2_O) in the surrounding could be the reasons why the ESPT would become kinetically feasible in the aggregated form of the **Py‐1** compound. Additionally, the ICT character shifting the charge density from the TPA‐OMe group towards the pyridine, even more effectively in the more polar environment of the water dispersion, would be responsible for making the pyridine unit a stronger base upon photoexcitation, thus facilitating the ESPT. In this context, one can speculate that the ICT would assist the ESPT phenomenon. To better emphasize the excited‐state re‐equilibration, the ultrafast dynamic of **Py‐1** nanoaggregates in DMF/buffer at pH=11, 5 and 1 were tentatively studied by fs‐TA and fs‐FUC spectroscopies. Unluckily, the signal‐to‐noise ratio of these measurements was terribly affected by scattering as for the case of fs‐TA and by the upconverted blue emission of the pump as for the fs‐FUC experiments. However, in the fs‐TA spectra (Figure S15) recorded at ca. 800 fs after excitation, one can note the double emission from both **Py‐1** at λ<500 nm and **Py‐1(H^+^)** at 650 nm at pH=5 (red line, pK_a_<pH<pK_a_*), duly assigned by comparison with the SE signals obtained at pH=11 (green line, pH>pK_a_*) and pH=1 (black line, pH<pK_a_). Additionally, at pH=5, some spectral signature of the ESA band of the protonated species becomes apparent at the red edge of the spectral window at λ>700 nm (Figure S15). These results differ from what observed for the monomers (Figure S7) where the excited state dynamics at pH=9 and 4.86 were almost identical in the absence of ESPT. The hinted double emission found in the case of the **Py‐1** aggregates, instead, might be consistent with the partial re‐equilibration upon excitation of the neutral form at pH=5, comprised between the pK_a_ and pK_a_* values.

## Conclusions

To sum up, the sensitivity to pH changes of three push‐pull pyridine derivatives bearing dimethoxytriphenylamine (TPA‐OMe) as electron donor and pyridine as electron acceptor, with different *ortho*‐functionalizations (−H, **Py‐1**, −Br, **Py‐2**, and −TPA‐OMe, **Py‐3**), was carefully gazed through steady‐state and time‐resolved (with nanosecond and femtosecond time resolution) spectroscopic techniques. Experiments were performed firstly for monomers in DMSO/water dilute solutions and, then, for aggregates of the compounds produced in water dispersions. Upon protonation of the pyridinic nitrogen, a significant red shift of the absorption and emission spectra, rationalized by the enhanced intramolecular charge transfer character of the singlet excited state, justified the acidochromic (from colorless to yellow) and acido(fluoro)chromic (from cyan to pink) behaviours observed for the investigated samples. The spectrophotometric and fluorimetric titrations carried out over a wide pH range allowed for the determination of the pK_a_ values resulting to be ca. 3.2 in the case of **Py‐1** and **Py‐3** and lower than 1 for **Py‐2**, due to the inductive effect of the bromine atom, when dissolved in dilute DMSO/water solution. Even though the Förster‐Weller cycle pointed out these push‐pull pyridines as potential photobases, the re‐equilibration in the singlet excited state was not observed for the isolated monomers in DMSO/water mixture, possibly due to the extremely short singlet lifetimes (tens/hundreds of ps). On the contrary, appealing properties emerged when combining the Aggregation‐Induced Emission capability of the investigated molecules to their pH‐sensitivity. As a matter of fact, the aggregation phenomenon in aqueous environment proved beneficial for a large variety of applications. Firstly, an increased basicity of the investigated pyridines was detected being the pK_a_ values raised from 3 to 4.3, towards the *optimum* for probing the intracellular pH variation for tumor cells recognition, indicating these pyridine‐like derivatives as potential biosensors. Additionally, the increased emission efficiency due to the AIE assured bright green fluorescence of the neutral forms and non‐negligible fluorescence in the red region of the Vis spectrum for the protonated species, suggesting these compounds as color‐tunable fluorescent probes. Moreover, the restricted aggregate environment in water suspensions was found to lengthen the singlet lifetimes. As a consequence, the slower deactivation rates together with the excess of water molecules in the surroundings allowed the Excited‐State Proton Transfer to be observed for the **Py‐1** aggregates. In fact, a pK_a_*=9.14 was measured through the fluorimetric titration in the case of **Py‐1**, highlighting the possible use of this molecule as photobase. The interplay of charge and proton transfers, ascribed to the multitasking nature of the pyridinic ring, acting both as electron and proton acceptor, was found to enable tunable multi‐responsive optical properties in the investigated push‐pull molecular architectures, thus becoming strategic building blocks for the development of new emissive photobases. In particular, the synergic activation of AIE and ESPT unveiled with this study via state‐of‐the‐art ultrafast spectroscopies not only constitutes an important step forward in the fundamental understanding of the photobehaviour of pyridine‐based fluorophores but also may allow the future rational design of optimized fluorescent biosensors.

## Experimental Section

### Chemicals

The synthesis of the investigated compounds (Scheme 1) was previously described.^[14]^ The current spectral and photophysical characterization was performed by employing organic solvents of spectroscopic grade and used as they were purchased: cyclohexane (CH, VWR Chemicals), dimethylformamide (DMF, ACS Reagent, Sigma‐Aldrich), dimethyl sulfoxide (DMSO, Carlo Erba). The different pHs were obtained by use of commercial buffer solutions (Pan‐Reac AppliChem by ITW Reagents) in the pH range 4–11, while HClO_4_ acid (70 %) was diluted in pure deionized water (H_2_O, PURELAB option, ELGA) to reach pH<4, down to H_0_=−5.00.

### Photophysical Measurements

The absorption spectra of the compounds in solution (∼1×10^−5^ M) were recorded by using a Cary 4E (Varian) spectrophotometer. Acid‐base titrations of the pyridine substrates required DMSO/Water mixture 60/40 %v/v for solubility issues. As reported in ref.,^[14]^ the photobehaviour of the investigated pyridines under these experimental conditions still refers to well‐dispersed monomers for those mixtures in which the water amount is less than 80 %. The impact of such percentage of the strongly basic DMSO on to the pH value of the buffer solution has been evaluated by the direct measurement of the [H_3_O^+^] concentration by means of a refillable Ag/AgCl pH electrode (Thermo Fisher Scientific) and compared to that of the pure buffer within the 2<pH<11 range. In this way, an almost perfect line correlation was obtained (Figure S1 in the Supporting Information) and thus used as calibration curve to apply the correction over the entire investigated pH interval. For the pH effect on aggregated species, a concentrated DMF solution (∼1×10^−3^ M) of the compounds was added to the buffer solution at different pHs to give a 1/99 %v/v mixture. The mixture was stirred on an ultrasonic bath to favour the dispersion and the aggregates formation. Fluorescence and excitation spectra were recorded by FS5 spectrofluorometer (Edinburgh Instruments) with the appropriate instrumental response corrections. The fluorescence quantum yields (φ_F_, experimental error±10 %) of dilute solutions/dispersions (A<0.1 at λ_exc_) were obtained by exciting each sample at the relative maximum absorption wavelength and by employing 9,10‐diphenylanthracene (φ_F_=0.73) and/or tetracene (φ_F_=0.17) in air‐equilibrated CH solution as reference compounds. The pK_a_ of the acid–base equilibria, as well as the concentration profiles and the spectra of intermediate species, are given considering the global fitting of multivariate spectrophotometric data, carried out by the ReactLab Equilibria software (Jplus Consulting). The parameters sum‐of‐squares (ssq) and deviation standard for the residuals (σ_r_) were used to evaluate the goodness of the fits. Fluorescence lifetimes were measured using the time‐correlated single‐photon counting (TC‐SPC) technique through an Edinburgh Instrument FS5 spectrofluorometer, equipped with an LED source centered at 375 nm, with a 0.5 ns temporal resolution. The experimental setup for the ultrafast transient absorption and fluorescence up conversion measurements has been widely described elsewhere.[^65^, ^66^, ^67^] The 400 nm excitation pulses, pump, (ca. 40 fs) are generated by a Spectra Physics amplified Ti:Sapphire laser and a Second Harmonic Generator (Apollo, Ultrafast Systems). Probe pulses are generated in the Helios transient absorption spectrometer (Ultrafast System) by passing a small portion of the 800‐nm light through an optical delay line (time window of 3.2 ns) and focusing it onto a Sapphire crystal (2 mm thick) to generate white‐light in the 450–800 nm spectral range. The transmitted signal is detected by a CCD camera with a temporal resolution of about 150 fs and a spectral resolution of 1.5 nm. In the UpConversion setup (Halcyone, Ultrafast System), the 400‐nm pulse excites the sample, whereas the fundamental laser beam (800 nm) acts as the “gate” light, after passing through a delay line. The fluorescence of the sample is then collected and focused onto a Barium Borate Oxide (BBO) crystal, set on a rotational stage, together with the delayed gate beam to realize the sum‐frequency generation phenomenon. A CCD detects the up‐converted fluorescence. Movements of the crystal through the rotational stage allow for broadband detection of the emission at each delay and thus acquisition of the entire time‐resolved fluorescence spectra. The time resolution is about 200 fs, while the spectral resolution is 1.5 nm. All the measurements were carried out under the magic angle condition in a cell (optical path=2 mm) with sample concentration so that 0.5<A<1 at 400 nm (pump wavelength). The solution was stirred during the experiments to avoid photoproduct interferences. The absorption spectra were double‐checked before and after each time‐resolved measurement to verify any relevant photodegradation. Anyway, no significant changes were observed. The experimental 3D data matrixes were analysed by performing the global analysis by Glotaran software.^[68]^

### Evaluation of pK_a_*

In those chemical systems where no equilibration in the excited state can be directly experimentally observed, the pK_a_ values for the first electronic excited state (pK_a_*) could still be approximately estimated by the indirect method proposed by Förster and Weller[^69^, ^70^, ^71^, ^72^, ^73^, ^74^, ^75^] reported in Equation 1:
(1)

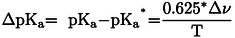




According to the Förster‐Weller cycle, the pK_a_* can be simply derived from the pK_a_ in the ground state and the difference (Δν) between the 0–0 energy of the protonated, Py−X(H^+^), and neutral, Py−X, species (where X=1, 2, 3), respectively. The Δν (in cm^−1^) is evaluated as the point of intersection of the normalized absorption and fluorescence spectra of the two species (Figures S6 and S12). Besides its ease of use, it has to be stressed out that one of the main approximations of the Förster‐Weller model is that it does not account for the possible reversibility of the proton transfer process and neglects the difference in the vibrational patterns of the electronic ground and excited states.

### Quantum Mechanical Calculations

Quantum‐mechanical calculations were carried out on the investigated molecules in their neutral and protonated forms using the Gaussian 09 package.^[76]^ Density functional theory (DFT) based on the B3LYP method was used to optimize the geometry and based on the CAM−B3LYP method to obtain the properties of the substrates in the ground state. Furthermore, the relaxed excited states S_1_ were optimized by B3LYP and characterized by time dependent (TD), by the CAM−B3LYP method.^[77]^ The Frank‐Condon excited singlet state were characterized by TD‐DFT CAM−B3LYP excited‐state calculations. In all cases, the 6–31+G(d,p) basis set was employed. Natural Transition Orbital (NTO) analysis was performed by employing the same method.[^78^, ^79^] Solvation effects induced by water were included in the calculations by means of the Conductor‐like Polarizable Continuum Model (CPCM).^[80]^

## Conflict of Interests

The authors declare no competing financial interest.

1

## Supporting information

As a service to our authors and readers, this journal provides supporting information supplied by the authors. Such materials are peer reviewed and may be re‐organized for online delivery, but are not copy‐edited or typeset. Technical support issues arising from supporting information (other than missing files) should be addressed to the authors.

Supporting Information

## Data Availability

The data that support the findings of this study are available from the corresponding author upon reasonable request.
