# Supplementary material for: Unlocking the Potential of Push‐Pull Pyridinic Photobases: Aggregation‐Induced Excited‐State Proton Transfer
Source: Chemistry. 2024 Nov 25;31(4):e202403388. doi: 10.1002/chem.202403388 (PMC11739827; doi:10.1002/chem.202403388)
Supplement: Supplementary file 1 — Supporting Information [file CHEM-31-e202403388-s001.pdf]

Chemistry–A European Journal

Supporting Information

Unlocking the Potential of Push-Pull Pyridinic Photobases: Aggregation-Induced Excited-State Proton Transfer

Letizia Mencaroni, Tommaso Bianconi, Maria Aurora Mancuso, Manju Sheokand, Fausto Elisei, Rajneesh Misra,* and Benedetta Carlotti*

Unlocking the Potential of Push-Pull Pyridinic Photobases: Aggregation-Induced Excited-State Proton Transfer

Letizia Mencaroni,[a] Tommaso Bianconi,[a] Maria Aurora Mancuso,[c] Manju Sheokand,[d] Fausto Elisei,[a] Rajneesh Misra*,[d] and Benedetta Carlotti*,[a]

[a] Dr. L. Mencaroni, Prof. F. Elisei, Prof. B. Carlotti
Department of Chemistry, Biology and Biotechnology and CEMIN
University of Perugia
06123 Perugia, Italy
E-mail: benedetta.carlotti@unipg.it

[b] Dr. T. Bianconi
Department of Chemistry
University of Wisconsin-Madison
53706 Madison, USA

[c] M.A. Mancuso
Istituto di Tecnologie Avanzate per l'Energia ''Nicola Giordano'' (CNR-ITAE)
98126 Messina, Italy

[d] M. Sheokand, Prof. R. Misra
Department of Chemistry
Indian Institute of Technology
453552 Indore, India
E-mail: rajneeshmisra@iiti.ac.in

List of Contents

S1. Calibration curve ______ 3

S2. Acid-base properties in solution ______ 4

S3. Ultrafast dynamic in solution ______ 7

S4. Acid-Base properties of aggregated species ______ 11

S5. Ultrafast dynamic of aggregated species ______ 13

S6. ReactLab Equilibria results ______ 14

S7. Quantum mechanical calculations ______ 18

S1. Calibration curve

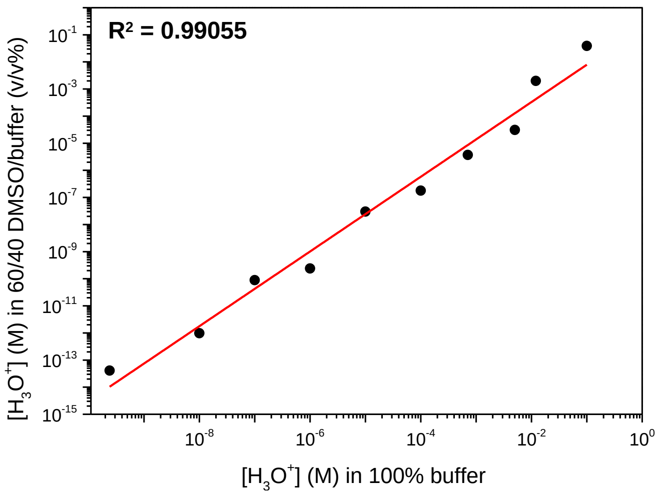

Figure S1: pH values measured in DMSO/buffer (60/40 %v/v) mixtures *vs* pH values measured for the commercial buffers (100% Water). The fitted line (red) was then used as calibration curve for the entire investigated pH range.

S2. Acid-base properties in solution

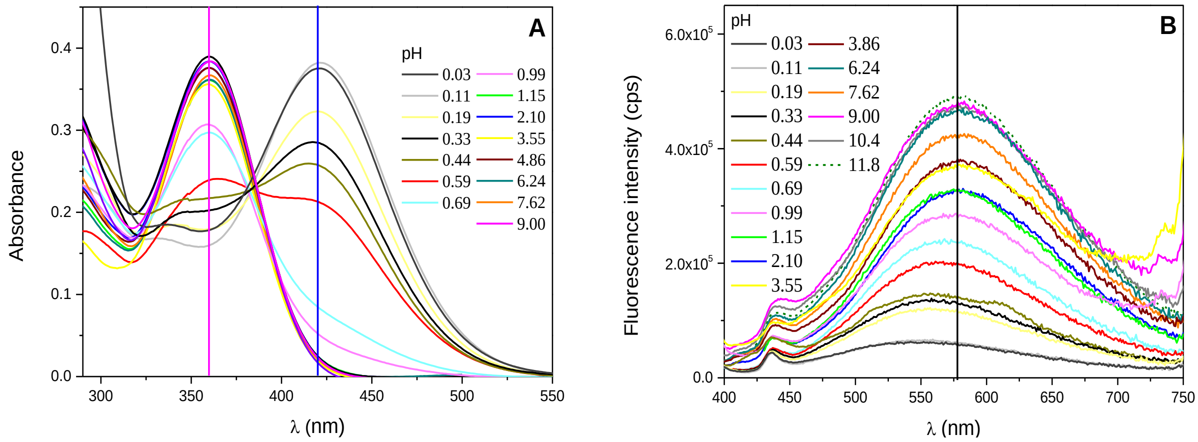

Figure S2: Spectrophotometric (A) and fluorimetric (B) titrations of compound **Py-2** in a large pH range (DMSO/buffer 60/40 %v/v). Fluorescence spectra were obtained by exciting each sample at the isosbestic point (λ_{exc} = 384 nm).

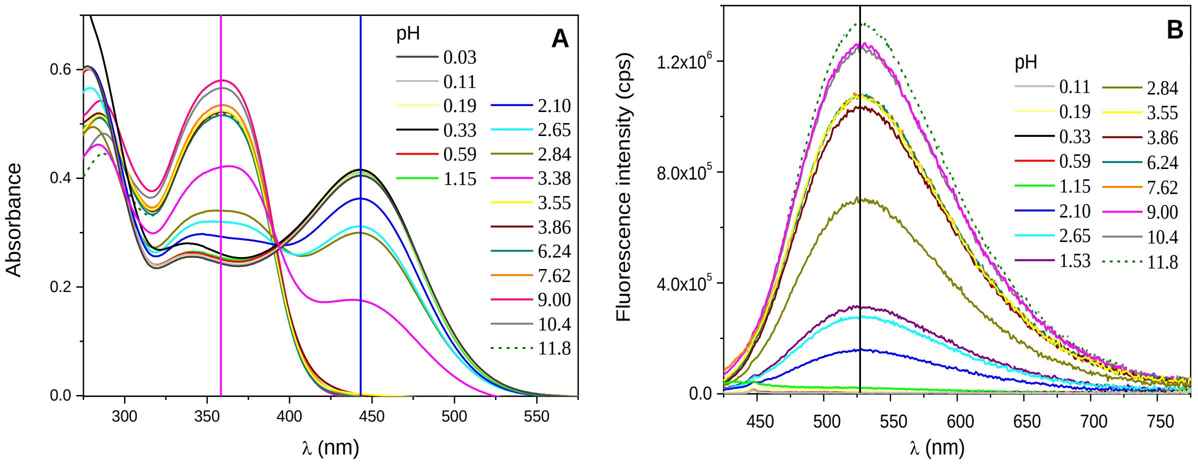

Figure S3: Spectrophotometric (A) and fluorimetric (B) titrations of compound **Py-3** in a large pH range (DMSO/buffer 70/30 %v/v). Fluorescence spectra were obtained by exciting each sample at the isosbestic point (λ_{exc} = 396 nm).

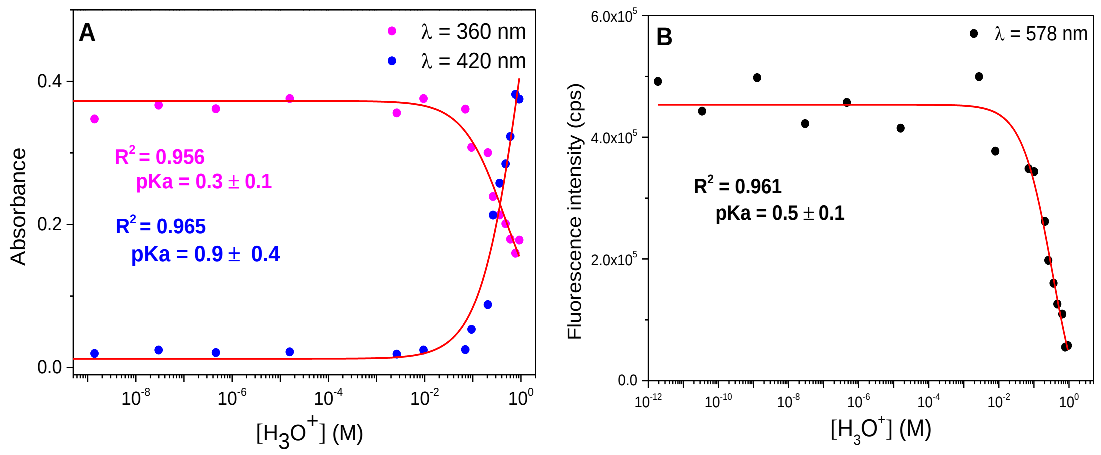

Figure S4: pK_a fittings obtained from the spectrophotometric (A) and fluorimetric (B) titrations for compound **Py-2** in DMSO/buffer 60/40 (%v/v).

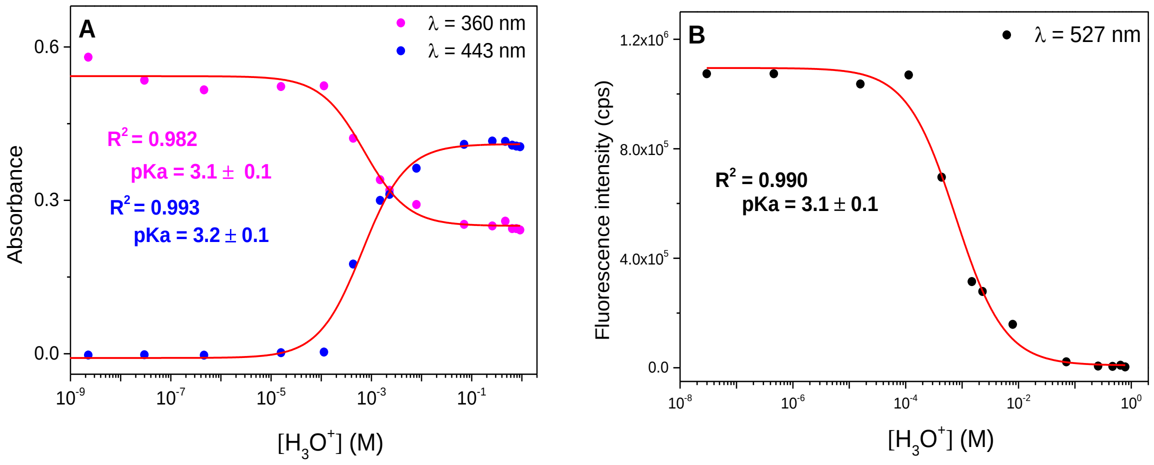

Figure S5: pK_a fittings obtained from the spectrophotometric (A) and fluorimetric (B) titrations for compound **Py-3** in DMSO/buffer 70/30 (%v/v).

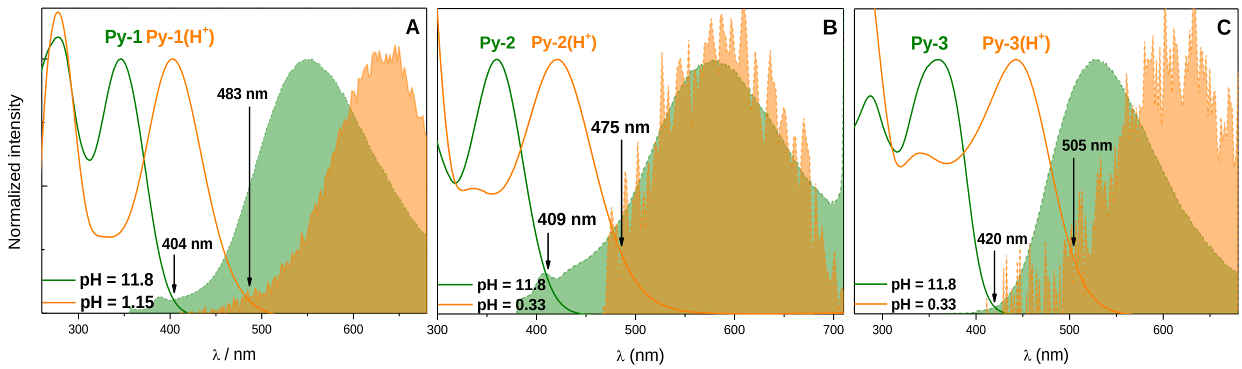

Figure S6: Normalized absorption (solid line) and emission (shaded area) spectra of the neutral and protonated species of **Py-1** (A), **Py-2** (B) and **Py-3** (C) in DMSO/buffer mixtures (60/40 %v/v) at different pHs. The intersection points between the normalized absorption and emission spectra of the neutral and protonated species used for the Förster-Weller cycle calculation are also highlighted.

Table S1: Fluorescence quantum yields measured for the investigated compounds in DMSO/buffer 60/40 %v/v mixtures at different pHs by employing different reference compounds (9,10-diphenylanthracene, DPA, *vs.* Tetracene).

	DPA			Tetracene		
pH/H_0	**Py-1**	**Py-2**	**Py-3**	**Py-1**	**Py-2**	**Py-3**
11.8	0.02	----	0.20	0.03	----	----
9.00	0.02	0.03	-----	0.03	0.03	----
4.86	0.02	0.02	0.20	0.03	0.02	----
1.15	0.0003	----	----	0.0004	----	----
0.33	----	0.003	----	----	0.004	0.003
0.19	---	----	----	----	----	0.004
0.03	----	0.003	----	----	0.003	----

S3. Ultrafast dynamic in solution

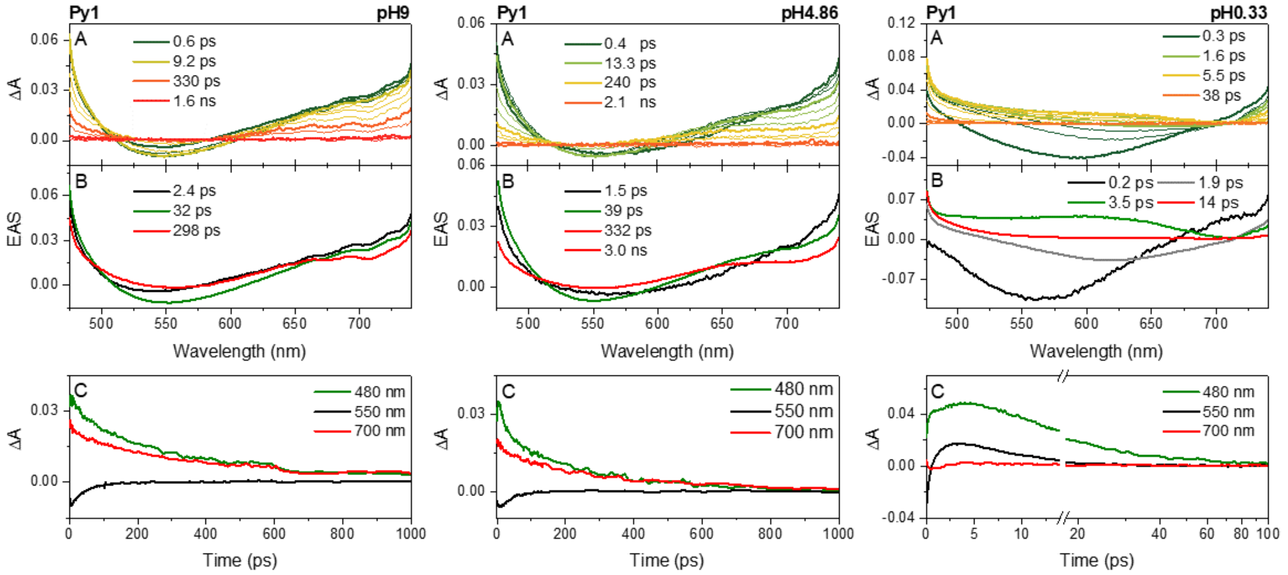

Figure S7: Femtosecond transient absorption (λ_{exc} = 400 nm) of **Py-1** in DMSO/buffer 60/40 (%v/v) at different pHs. Panel A: spectra obtained at different delay times after excitation. Thicker lines refer to representative spectra corresponding to the explicit time delays (see legend). Panel B: Evolution Associated Spectra (EAS) obtained for the transients provided by Global Analysis with related lifetimes: Solv./$S_{1,LE}$ (solvation/locally-excited singlet state, black line), Solv. (diffusive solvation, gray line), Solv./VC (solvation/vibrational cooling, brown line), SR (structural relaxation, green line) and $S_{1,ICT}$, (stabilized singlet state with ICT nature, red line). Panel C: Representative kinetics acquired at selected wavelengths to probe the $S_{1,LE}$, black, and $S_{1,ICT}$, red, deactivation dynamics.

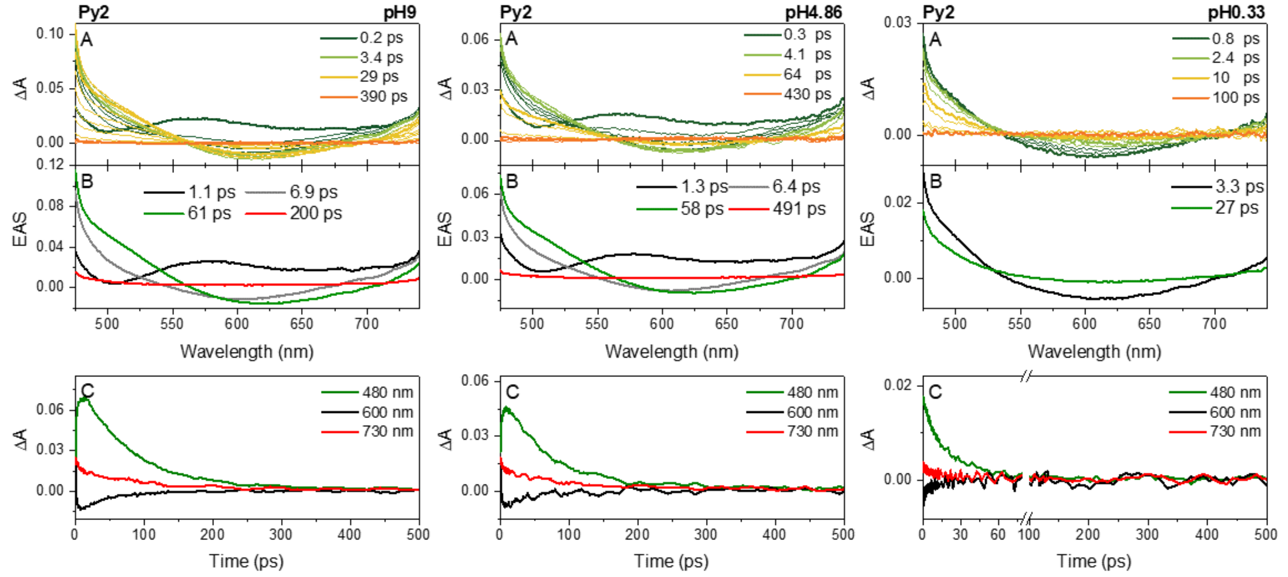

Figure S8: Femtosecond transient absorption (λ_{exc} = 400 nm) of **Py-2** in DMSO/buffer 60/40 (%v/v) at different pHs. Panel A: spectra obtained at different delay times after excitation. Thicker lines refer to representative spectra corresponding to the explicit time delays (see legend). Panel B: Evolution Associated Spectra (EAS) obtained for the transients provided by Global Analysis with related lifetimes: Solv./$S_{1,LE}$ (solvation/locally-excited singlet state, black line), Solv./VC (solvation/vibrational cooling, brown line), SR (structural relaxation, green line) and $S_{1,ICT}$, (stabilized singlet state with ICT nature, red line). Panel C: Representative kinetics acquired at selected wavelengths to probe the $S_{1,LE}$, black, and $S_{1,ICT}$, red, deactivation dynamics.

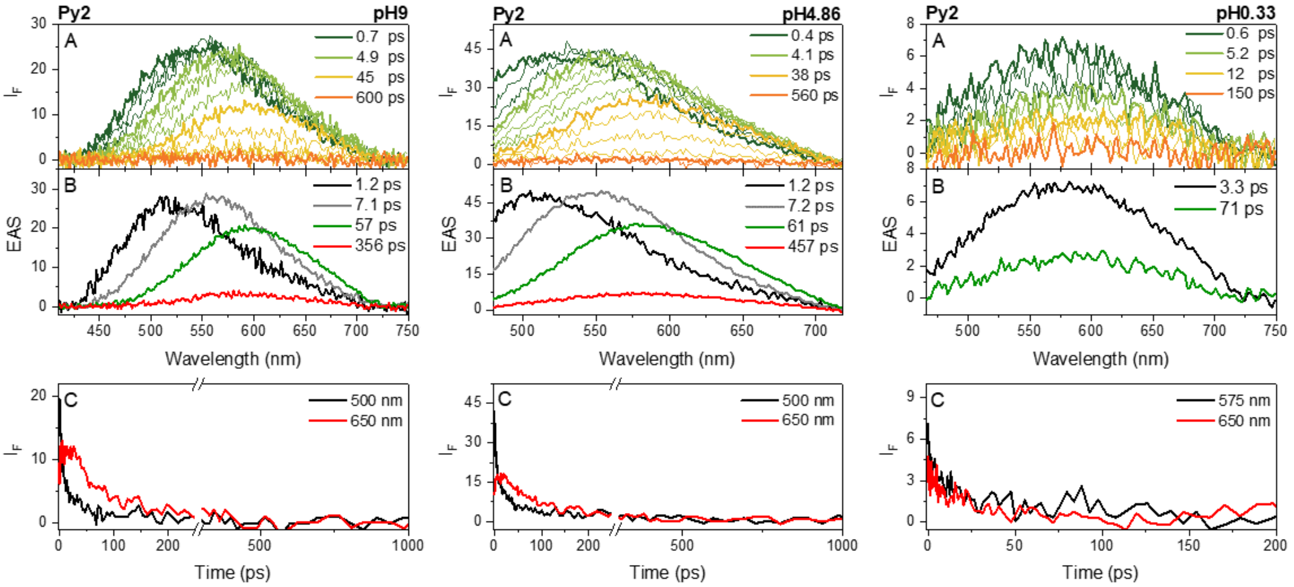

Figure S9: Femtosecond fluorescence up conversion (λ_{exc} = 400 nm) of **Py-2** in DMSO/buffer 60/40 (%v/v) at different pHs. Panel A: spectra obtained at different delay times after excitation. Thicker lines refer to representative spectra corresponding to the explicit time delays (see legend). Panel B: Evolution Associated Spectra (EAS) obtained for the transients provided by Global Analysis with related lifetimes: Solv./$S_{1,LE}$ (solvation/locally-excited singlet state, black line), Solv./VC (solvation/vibrational cooling, brown line), SR (structural relaxation, green line) and $S_{1,ICT}$, (stabilized singlet state with ICT nature, red line). Panel C: Representative kinetics acquired at selected wavelengths to probe the $S_{1,LE}$, black, and $S_{1,ICT}$, red, deactivation dynamics.

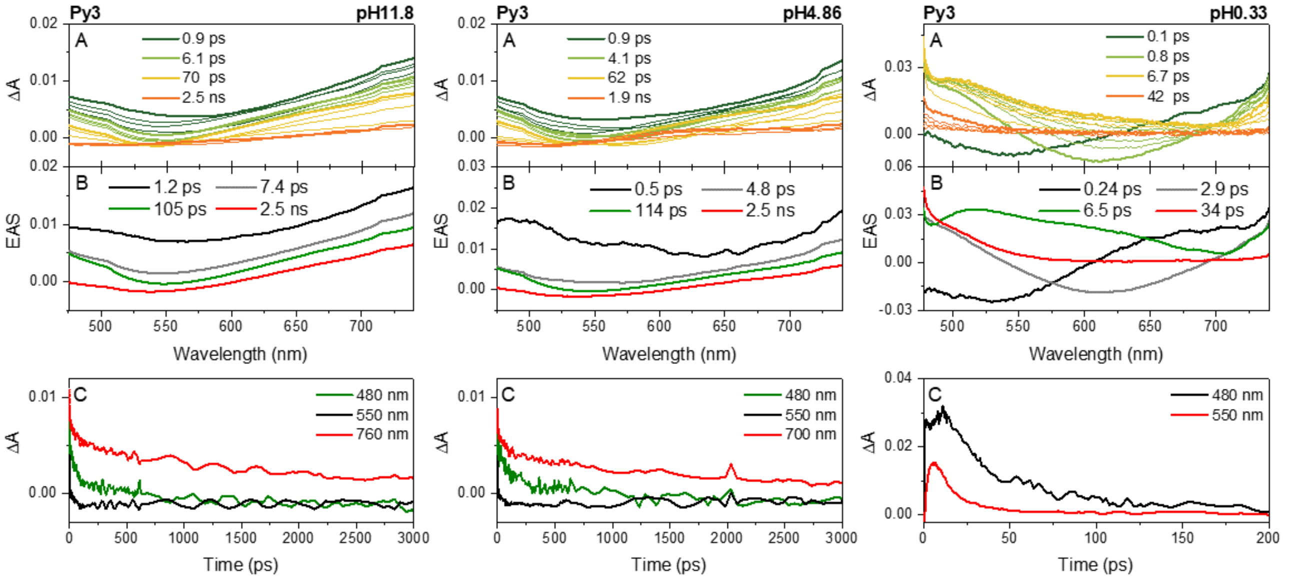

Figure S10: Femtosecond transient absorption (λ_{exc} = 400 nm) of **Py-3** in DMSO/buffer 60/40 (%v/v) at different pHs. Panel A: spectra obtained at different delay times after excitation. Thicker lines refer to representative spectra corresponding to the explicit time delays (see legend). Panel B: Evolution Associated Spectra (EAS) obtained for the transients provided by Global Analysis with related lifetimes: Solv./$S_{1,LE}$ (solvation/locally-excited singlet state, black line), Solv. (diffusive solvation, gray line), Solv./VC (solvation/vibrational cooling, brown line), SR (structural relaxation, green line) and $S_{1,ICT}$, (stabilized singlet state with ICT nature, red line). Panel C: Representative kinetics acquired at selected wavelengths to probe the $S_{1,LE}$, black, and $S_{1,ICT}$, red, deactivation dynamics.

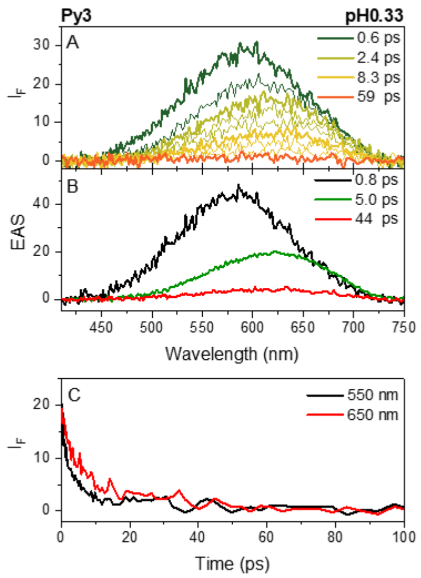

Figure S11: Femtosecond fluorescence up conversion (λ_{exc} = 400 nm) of **Py-3** in DMSO/buffer 60/40 (%v/v) at different pHs. Panel A: spectra obtained at different delay times after excitation. Thicker lines refer to representative spectra corresponding to the explicit time delays (see legend). Panel B: Evolution Associated Spectra (EAS) obtained for the transients provided by Global Analysis with related lifetimes: Solv./$S_{1,LE}$ (solvation/locally-excited singlet state, black line), Solv./VC (solvation/vibrational cooling, brown line), SR (structural relaxation, green line) and $S_{1,ICT}$, (stabilized singlet state with ICT nature, red line). Panel C: Representative kinetics acquired at selected wavelengths to probe the $S_{1,LE}$, black, and $S_{1,ICT}$, red, deactivation dynamics.

Table S2: Results of Global Fit of the fs-FUC and fs-TA data (λ_{exc} = 400 nm) of compounds **Py-1**, **Py-2** and **Py-3** in DMSO/buffer (60/40 %v/v) solutions at different pHs.

	**Py-1**		**Py-2**		**Py-3**		
**pH**	τ_{FUC} / ps	τ_{TA} / ps	τ_{FUC} / ps	τ_{TA} / ps	τ_{FUC} / ps	τ_{TA} / ps	Assignment
9.00[a]	3.4	2.4	1.2	1.1		1.2	Solv./$S_{1,LE}$
			7.1	6.9		7.4	Solv./VC
	20	32	57	61		105	SR
	273	298	356	200		2500[b]	$S_{1,ICT}$ (n)
4.86	2.9	1.5	1.2	1.3		0.5	Solv./$S_{1,LE}$
			7.2	6.4		4.8	Solv./VC
	62	39	61	58		114	SR
	383	332	457	491		2500[b]	$S_{1,ICT}$ (n)
0.33	0.8	0.2			0.8	0.2	Solv./$S_{1,LE}$
		1.5				2.9	Solv.
	3.7	3.5	3.3	3.3	5.0	6.5	Solv./VC
	22	14	27	71	44	34	$S_{1,ICT}$ (H^+)

[a] fs-TA results for **Py-3** were acquired at pH = 11.8. [b]S_1, is a planar ICT states, as stated in ref. [14].

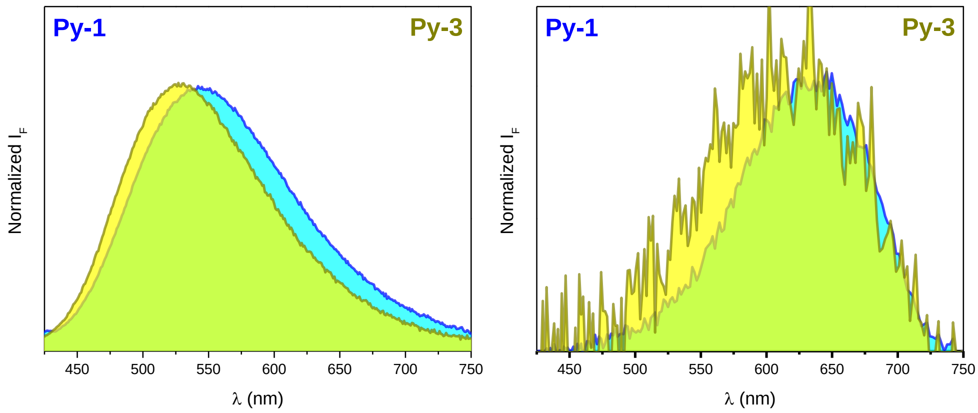

Figure S12: Normalized emission spectra assigned to the $S_{1,ICT}$ state for compounds **Py-1** (blue) and **Py-3** (yellow) at pH = 9 (left) and **Py-1(H$^+$)** (blue) and **Py-3(H$^+$)** (yellow) at pH = 0.33 (right).

S4. Acid-Base properties of aggregated species

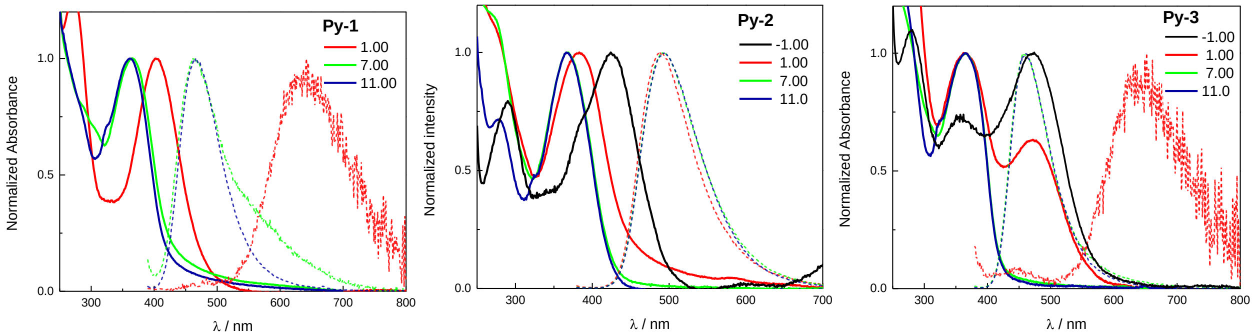

Figure S13. Absorption (solid) and emission (dashed) spectra of **Py-1-3** aggregates in water dispersions (DMF/buffer 1/99 %v/v mixtures) at H_0 = −1 and pH 1, 7 and 11. Fluorescence spectra were obtained by exciting at λ_{exc} = 380 nm in the case of **Py-1** and λ_{exc} = 370 nm for **Py-2** and **Py-3**, respectively.

Table S3: Acid-base properties of the investigated aggregates in water dispersion (DMF/buffer 1/99 %v/v), derived by spectrophotometric ($pK_{a,abs}$) and fluorimetric ($pK_{a,em}$) titrations analyzed by best fitting procedures through ReactLab Equilibira and predicted by ACD/Labs software ($pK_{a,calc}$). Last column shows the pK_a* values estimated by using the Förster cycle ($pK_a{}^*{}_{th}$).

Compound	λ_{abs} / nm	λ_{em} / nm	$pK_{a,abs}$	$pK_{a,em}$	$pK_{a,calc.}$	$pK_a{}^*{}_{th}$
**Py-1**	358	465	4.27 ± 0.01	4.25 ± 0.02	4.96; −4.57[a]	
**Py-1(H⁺)**	402	640		9.14 ± 0.02		12.4
**Py-2**	376	491			0.52; −5.61[a]	----
**Py-2(H⁺)**	423	ND				
**Py-3**	376	465			4.56; −4.45[a]; −5.45[a]	17.1
**Py-3(H⁺)**	470	645				

[a] the $pK_{a,2}$ values refer to the possible protonation of the triphenylamine unit, being two in the case of **Py-3**.

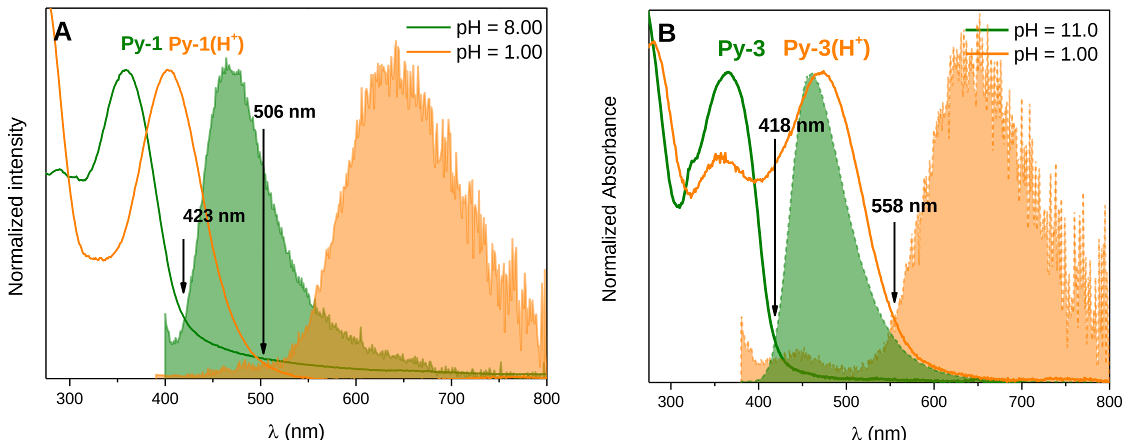

Figure S14: Normalized absorption (solid line) and emission (shaded area) spectra of the neutral and protonated species of **Py-1** (A) and **Py-3** (B) aggregates in water dispersion (DMF/buffer 1/99 %v/v) at different pHs.

Table S4. Photophysical properties of the aggregates in water dispersion (DMF/buffer 1/99 %v/v) at different pHs.

	**Py-1**				**Py-2**				**Py-3**			
pH	λ_{abs} / nm	λ_{em} / nm	τ_F / ns	ϕ_F	λ_{abs} / nm	λ_{em} / nm	τ_F / ns	ϕ_F	λ_{abs} / nm	λ_{em} / nm	τ_F / ns	ϕ_F
11	358	466	0.03 (19%)<br>1.1 (46%)<br>3.5 (35%)	0.23	368	493	< 0.5 (9%)<br>2.9 (58%)<br>7.1 (33%)	0.24	374	461	< 0.5 (26%)<br>1.3 (56%)<br>4.7 (17%)	0.11
7	365	465	0.05 (34%)<br>0.80 (41%)<br>2.5 (26%)	0.07	368	493	< 0.5 (9%)<br>2.7 (57%)<br>6.7 (34%)	0.21	374	461	< 0.5 (37%)<br>1.1 (52%)<br>4.2 (11%)	0.09
1	402	650	0.16 (53%)<br>0.65 (47%)	0.001	383	489	< 0.5 (16%)<br>1.9 (60%)<br>5.0 (24%)	0.12	374/<br>472	650	< 0.5 (69%)<br>1.0 (23%)<br>4.3 (8%)	0.004

S5. Ultrafast dynamic of aggregated species

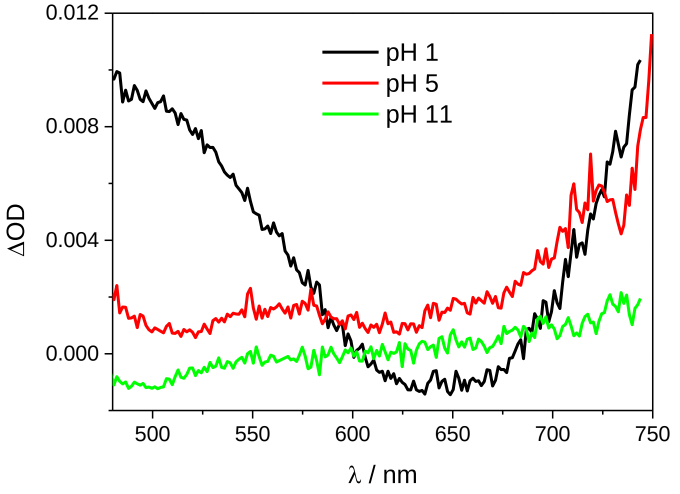

Figure S15: Representative spectra of **Py-1** aggregates in DMF/buffer (10/90 in %v/v) at different pHs (1, black line, 5, red line, and 11, green line) as obtained by fs-TA (λ_{exc} = 400 nm) at a delay time of 800 fs after photoexcitation.

S6. ReactLab Equilibria results

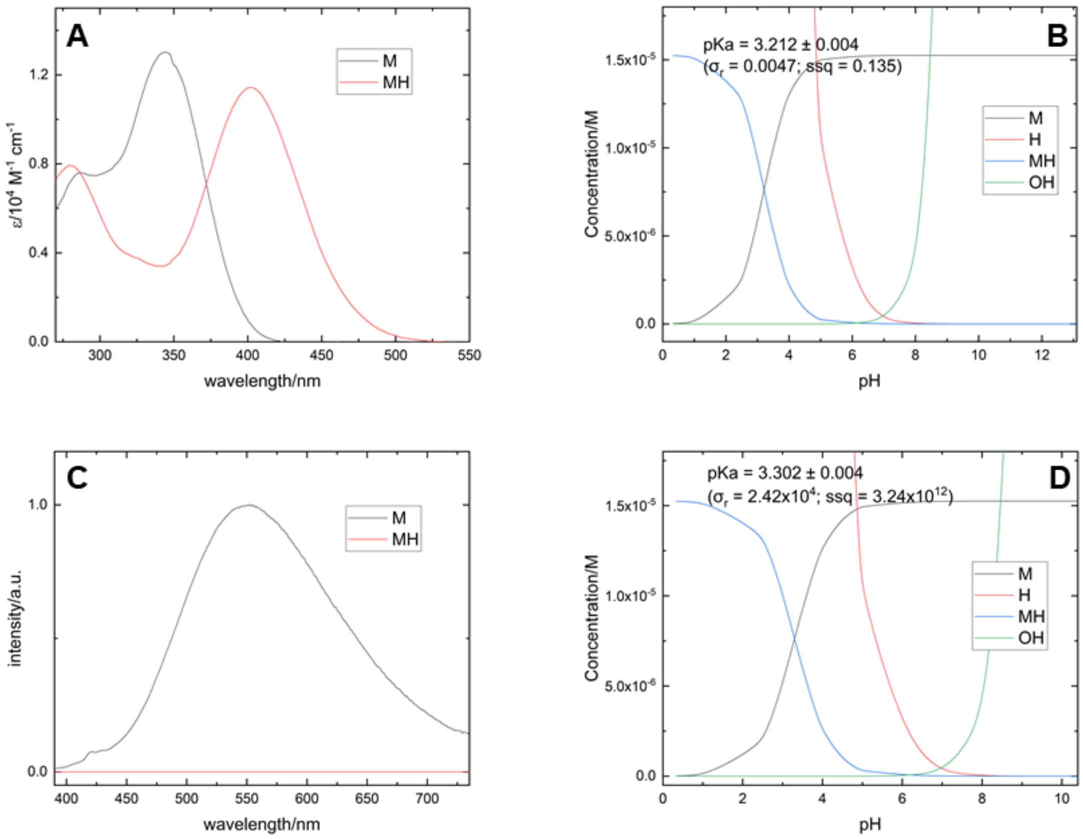

Figure S16: Quantitative absorption (panel A) and emission (panel C) spectra of the neutral (M) and protonated (MH) species of **Py-1** monomers together with their relative concentration profiles extracted from the global fitting through REACTLab Equilibria software in DMSO/buffer mixtures (60/40 %v/v) at different pHs.

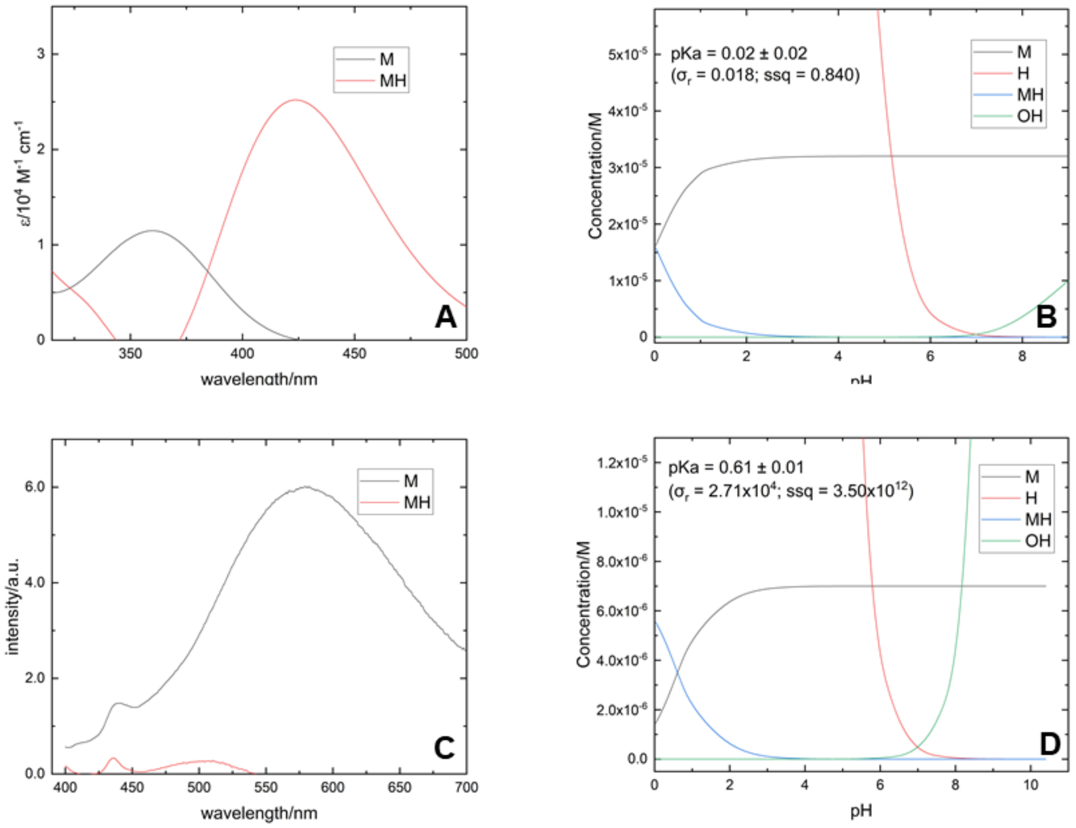

Figure S17: Quantitative absorption (panel A) and emission (panel C) spectra of the neutral (M) and protonated (MH) species of **Py-2** monomers together with their relative concentration profiles extracted from the global fitting through REACTLab Equilibria software in DMSO/buffer mixtures (60/40 %v/v) at different pHs.

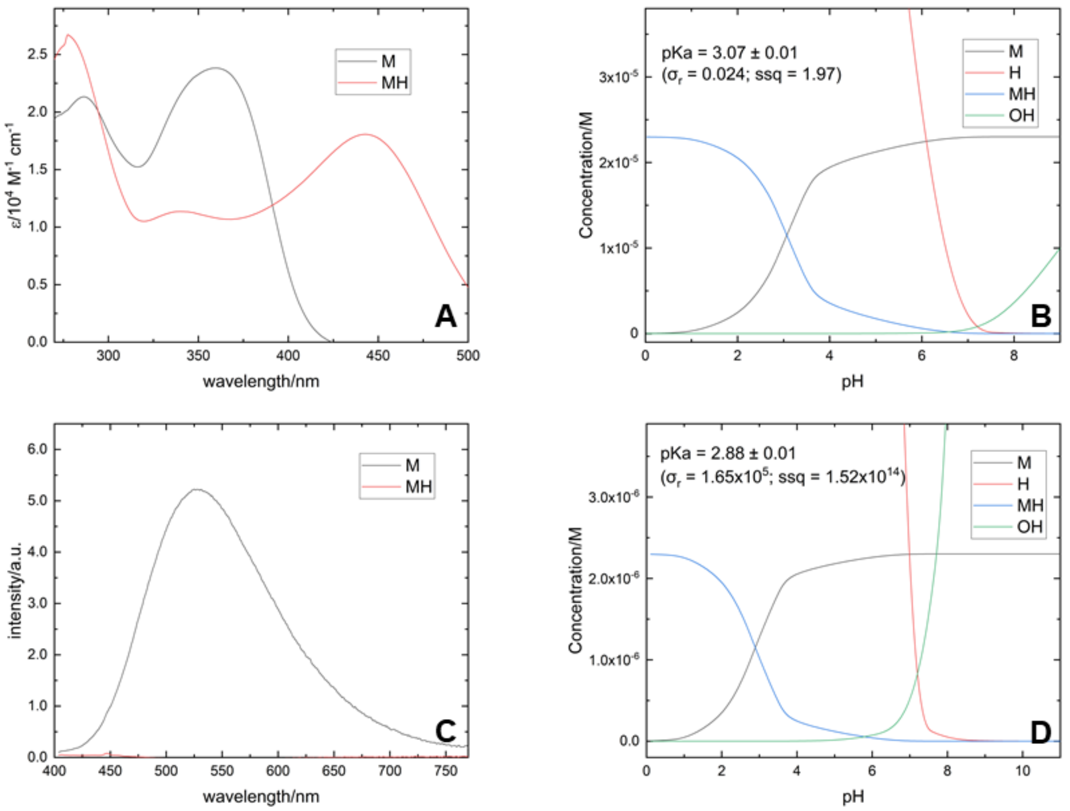

Figure S18: Quantitative absorption (panel A) and emission (panel C) spectra of the neutral (M) and protonated (MH) species of **Py-3** monomers together with their relative concentration profiles extracted from the global fitting through REACTLab Equilibria software in DMSO/buffer mixtures (60/40 %v/v) at different pHs.

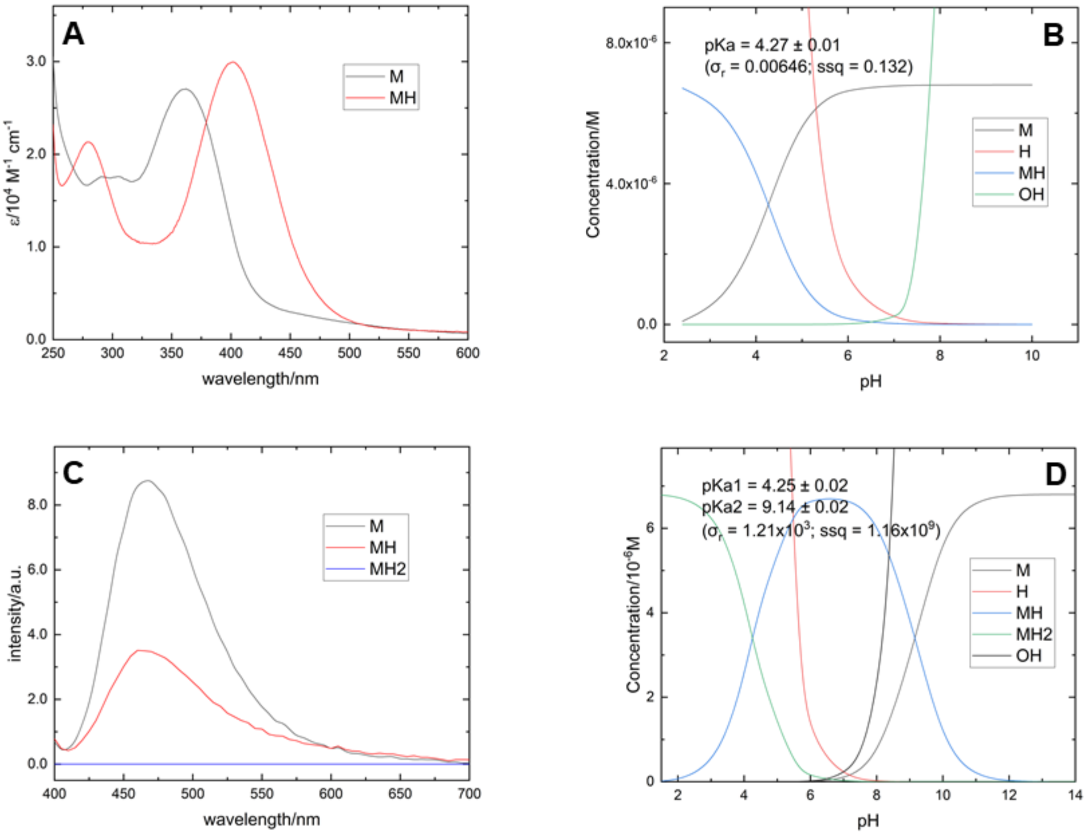

Figure S19: Quantitative absorption (panel A) and emission (panel C) spectra of the neutral (M) and protonated (MH) species of **Py-1** aggregates together with their relative concentration profiles extracted from the global fitting through REACTLab Equilibria software in DMF/buffer mixtures (1/99 %v/v) at different pHs.

S7. Quantum mechanical calculations

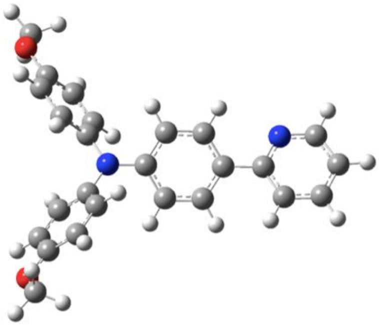

Py1@minS_0

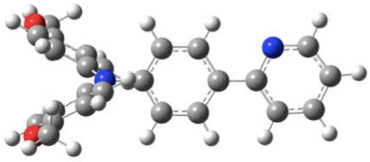

Py1@minS_1

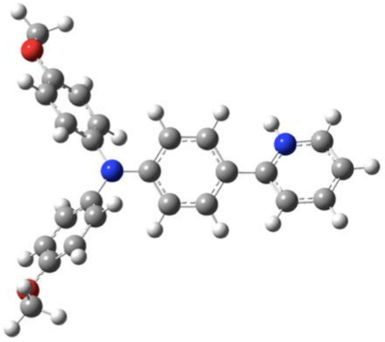

Py1H$^+$@minS_0

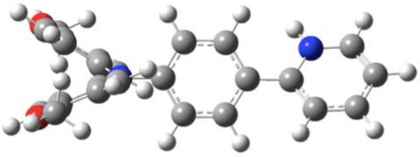

Py1 H$^+$@minS_1

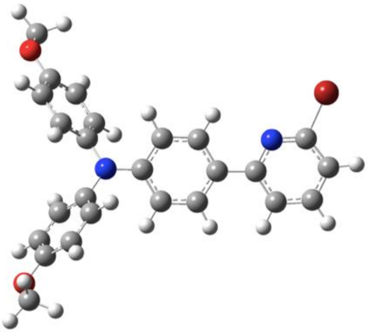

Py2@minS_0

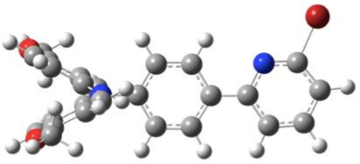

Py2@minS_1

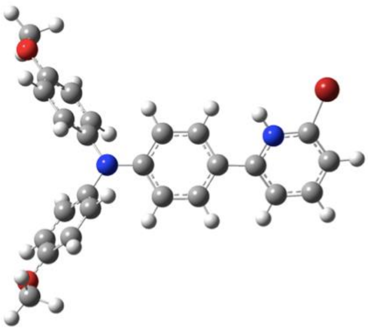

Py2H$^+$@minS_0

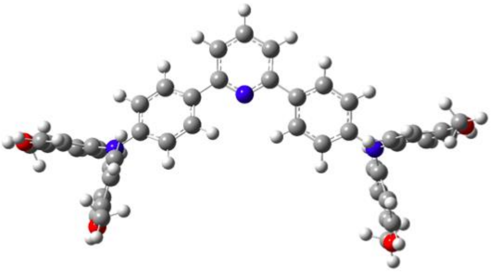

Py3@minS_0

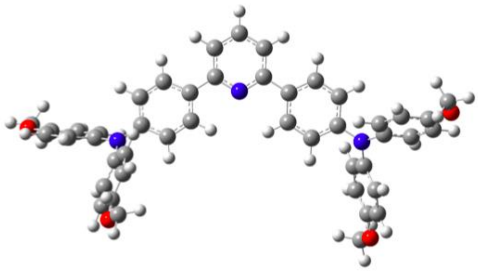

Py3@minS_1

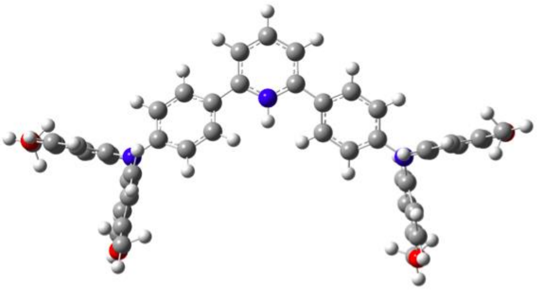

Py3H$^+$@minS_0

Figure S20: Ground state (minS_0) and excited state (minS_1) optimized geometries for the investigated neutral (**Py-1**, **Py-2**, **Py-3**) and protonated (**Py-1H$^+$**, **Py-2 H$^+$**, **Py-3 H$^+$**) compounds by B3LYP/6-31+G(d,p) in water.

Table S5: Absorption and emission wavelengths (λ), oscillator strength (f) and molecular orbitals of **Py-1** in water calculated by the CAM-B3LYP/6-31+G(d,p)//B3LYP/6-31+G(d,p) model, together with the experimental absorption maxima.

Transition	λ_{th}/nm	f	MO	%	λ_{exp}/nm
$S_0 \rightarrow T_1$	463	0.0000	$\pi_H \rightarrow \pi_L^*$	57	
$S_0 \rightarrow T_2$	395	0.0000	$\pi_H \rightarrow \pi_{L+3}^*$	24	
$S_0 \rightarrow T_3$	370	0.0000	$\pi_{H-4} \rightarrow \pi_{L+5}^*$<br>$\pi_{H-2} \rightarrow \pi_{L+3}^*$<br>$\pi_{H-1} \rightarrow \pi_L^*$	11<br>11<br>10	
$S_0 \rightarrow T_4$	351	0.0000	$\pi_H \rightarrow \pi_{L+1}^*$	55	
$S_0 \rightarrow S_1$	334	1.1731	$\pi_H \rightarrow \pi_L^*$	85	342
$S_0 \rightarrow T_5$	331	0.0000	$\pi_{H-8} \rightarrow \pi_{L+2}^*$<br>$\pi_{H-6} \rightarrow \pi_L^*$<br>$\pi_{H-6} \rightarrow \pi_{L+2}^*$	9<br>9<br>8	
$S_0 \rightarrow T_6$	322	0.0000	$\pi_H \rightarrow \pi_{L+5}^*$	56	
$S_0 \rightarrow T_7$	318	0.0000	$\pi_H \rightarrow \pi_{L+8}^*$	29	
$S_0 \rightarrow S_2$	312	0.0880	$\pi_H \rightarrow \pi_{L+1}^*$	75	
$S_0 \rightarrow T_8$	301	0.0000	$\pi_{H-1} \rightarrow \pi_{L+2}^*$	16	
$S_0 \rightarrow T_9$	292	0.0000	$\pi_{H-3} \rightarrow \pi_{L+1}^*$	22	
$S_0 \rightarrow T_{10}$	282	0.0000	$n_{H-7} \rightarrow \pi_L^*$	46	
$S_0 \rightarrow S_3$	279	0.4421	$\pi_H \rightarrow \pi_{L+3}^*$	56	
$S_0 \rightarrow S_4$	272	0.0311	$\pi_H \rightarrow \pi_{L+2}^*$<br>$\pi_H \rightarrow \pi_{L+5}^*$	20<br>23	
$S_0 \rightarrow S_5$	272	0.0426	$\pi_H \rightarrow \pi_{L+5}^*$	55	
$S_0 \rightarrow S_6$	258	0.0899	$\pi_H \rightarrow \pi_{L+8}^*$	18	
$S_0 \rightarrow S_7$	247	0.0078	$\pi_H \rightarrow \pi_{L+4}^*$	59	
$S_0 \rightarrow S_8$	245	0.0135	$n_{H-7} \rightarrow \pi_L^*$	64	
$S_0 \rightarrow S_9$	245	0.0632	$\pi_H \rightarrow \pi_{L+7}^*$	26	
$S_0 \rightarrow S_{10}$	229	0.0690	$\pi_{H-1} \rightarrow \pi_L^*$	34	
$S_1 \rightarrow S_0$	529	0.3336	$\pi_H \rightarrow \pi_L^*$	99	550

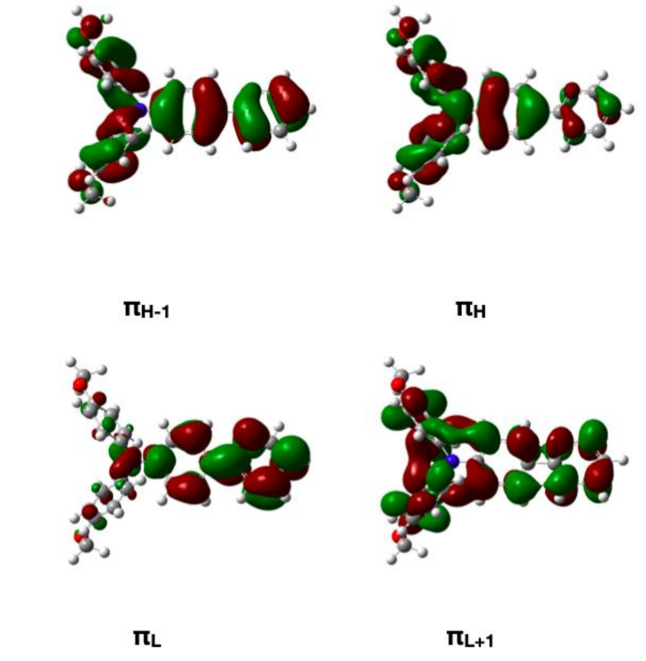

Figure S21: Frontier molecular orbitals of **Py-1** at minS$_0$ geometry.

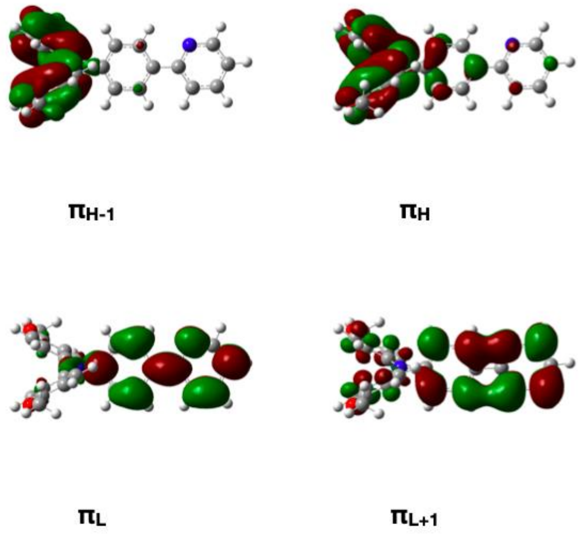

Figure S22: Frontier molecular orbitals of **Py-1** at minS$_1$ geometry.

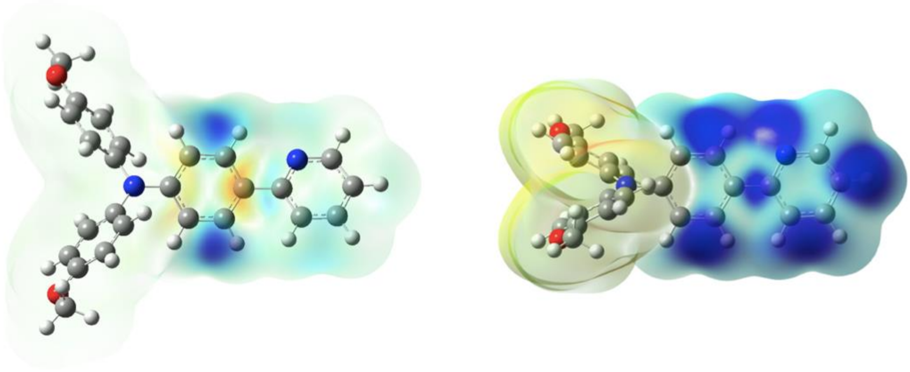

Figure S23: Effect of the S$_0$→S$_1$ transition on the electron density of **Py-1** at minS$_0$ (left) and minS$_1$ (right) geometries; increase and decrease of electron densities are represented by blue (+0.0001) and red (-0.0001), respectively.

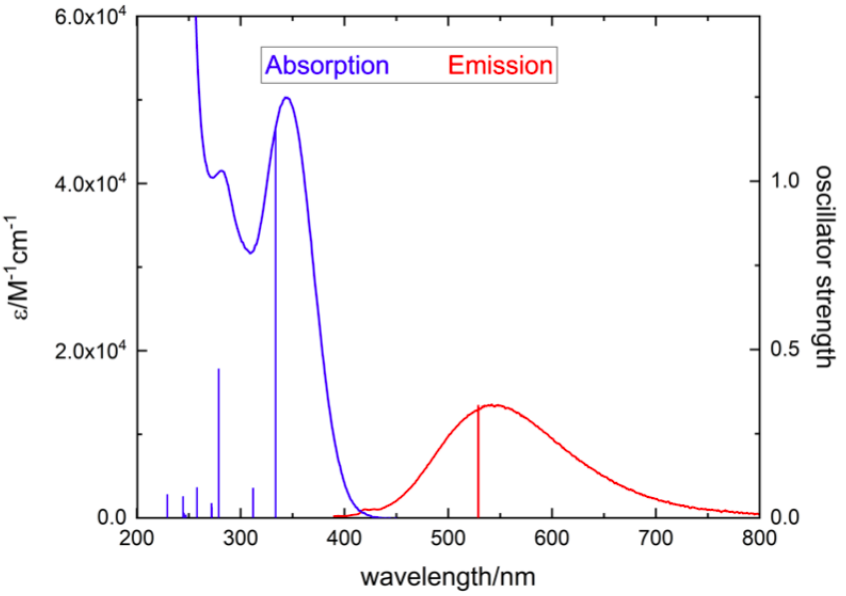

Figure S24: Absorption and emission spectra of **Py-1** (full lines) compared with those calculated by the CAM-B3LYP/6-31+G(d,p)//B3LYP/6-31+G(d,p) model (vertical bars) in water (CPCM).

Table S6: Absorption and emission wavelengths (λ), oscillator strength (f) and molecular orbitals of **Py-1H⁺** in water calculated by the CAM-B3LYP/6-31+G(d,p)//B3LYP/6-31+G(d,p) model, together with the experimental absorption maxima.

Transition	λ_{th}/nm	f	MO	%	λ_{exp}/nm
$S_0\rightarrow T_1$	518	0.0000	$\pi_H\rightarrow\pi_L^*$	61	
$S_0\rightarrow S_1$	401	1.1299	$\pi_H\rightarrow\pi_L^*$	82	402
$S_0\rightarrow T_2$	384	0.0000	$\pi_H\rightarrow\pi_{L+2}^*$<br>$\pi_H\rightarrow\pi_{L+3}^*$<br>$\pi_H\rightarrow\pi_{L+4}^*$	17<br>14<br>12	
$S_0\rightarrow T_3$	375	0.0000	$\pi_H\rightarrow\pi_{L+1}^*$	27	
$S_0\rightarrow T_4$	364	0.0000	$\pi_{H-2}\rightarrow\pi_{L+1}^*$	12	
$S_0\rightarrow T_5$	337	0.0000	$\pi_H\rightarrow\pi_{L+2}^*$	59	
$S_0\rightarrow T_6$	317	0.0000	$\pi_{H-6}\rightarrow\pi_L^*$	21	
$S_0\rightarrow T_7$	310	0.0000	$\pi_H\rightarrow\pi_{L+6}^*$	31	
$S_0\rightarrow T_8$	310	0.0000	$\pi_H\rightarrow\pi_{L+5}^*$	28	
$S_0\rightarrow T_9$	307	0.0000	$\pi_{H-5}\rightarrow\pi_{L+2}^*$	16	
$S_0\rightarrow S_2$	304	0.1070	$\pi_H\rightarrow\pi_{L+2}^*$	50	
$S_0\rightarrow S_3$	292	0.0802	$\pi_H\rightarrow\pi_{L+1}^*$<br>$\pi_H\rightarrow\pi_{L+2}^*$	42<br>38	
$S_0\rightarrow T_{10}$	286	0.0000	$\pi_{H-5}\rightarrow\pi_L^*$	53	
$S_0\rightarrow S_4$	266	0.3360	$\pi_H\rightarrow\pi_{L+4}^*$	27	
$S_0\rightarrow S_5$	264	0.1283	$\pi_{H-2}\rightarrow\pi_L^*$	44	
$S_0\rightarrow S_6$	263	0.0604	$\pi_H\rightarrow\pi_{L+5}^*$	60	
$S_0\rightarrow S_7$	262	0.1603	$\pi_H\rightarrow\pi_{L+6}^*$	48	
$S_0\rightarrow S_8$	246	0.0939	$\pi_{H-5}\rightarrow\pi_L^*$	33	
$S_0\rightarrow S_9$	243	0.0454	$\pi_H\rightarrow\pi_{L+7}^*$	57	
$S_0\rightarrow S_{10}$	240	0.1468	$\pi_{H-1}\rightarrow\pi_L^*$	62	
$S_1\rightarrow S_0$	689	0.1322	$\pi_H\rightarrow\pi_L^*$	90	625

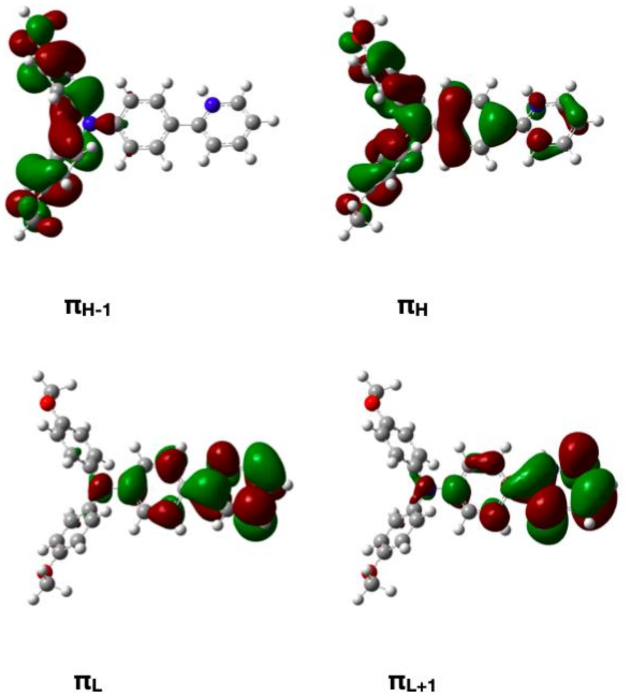

Figure S25: Frontier molecular orbitals of **Py-1H$^+$** at minS_0 geometry.

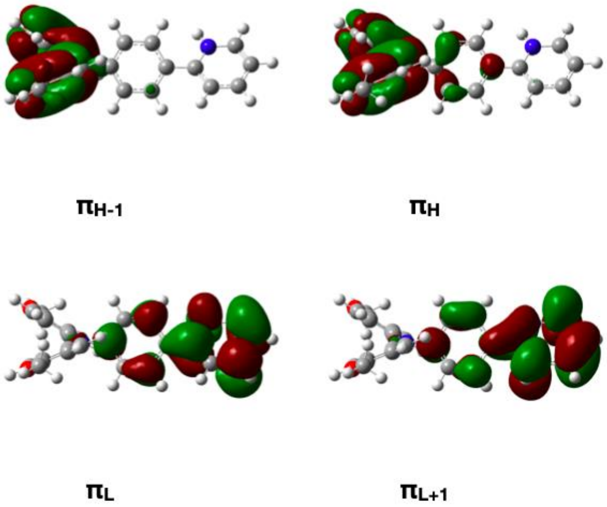

Figure S26: Frontier molecular orbitals of **Py-1H$^+$** at minS_1 geometry.

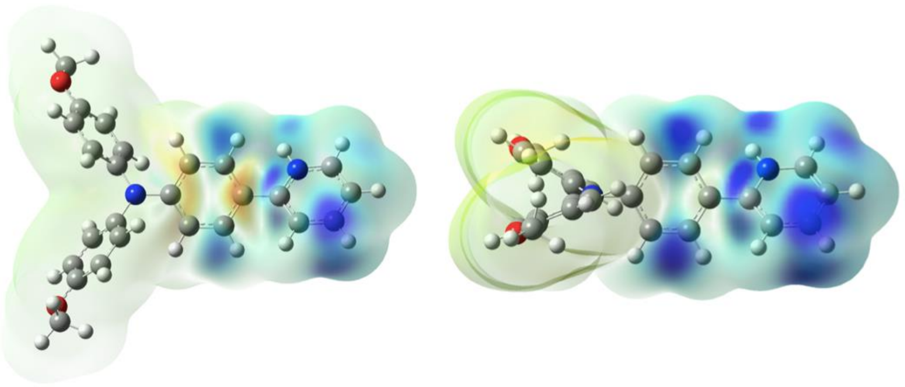

Figure S27: Effect of the $S_0 \rightarrow S_1$ transition on the electron density of **Py-1H$^+$** at minS_0 (left) and minS_1 (right) geometries; increase and decrease of electron densities are represented by blue (+0.0001) and red (-0.0001), respectively.

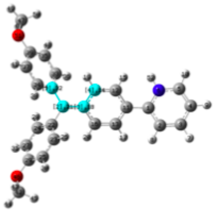

$\Theta C_{22}N_{21}C_{18}C_{14}$/deg	$E(S_0)$/eV	$E(S_0)$/kcal mol^{-1}	$E(S_1)$/eV	$E(S_1)$/kcal mol^{-1}	λ_{em}/nm
**10**	0.0087	0.2000	2.9118	67.0894	574
**20**	**0.0000**	0.0000	2.8204	64.9842	594
**30**	0.0066	0.1518	2.7532	63.4357	617
**40**	0.0294	0.6765	2.7050	62.3256	673
**50**	0.0626	1.4421	2.6788	61.7214	664
**60**	0.1049	2.4170	**2.6691**	61.4975	689
**70**	0.1572	3.6213	2.6721	61.5673	714
**80**	0.2107	4.8554	2.6898	61.9753	739
**90**	0.2769	6.3788	2.7200	62.6700	758
**100**	0.3787	8.7255	2.7668	63.7490	770
**110**	0.5153	11.8738	2.8346	65.3121	763

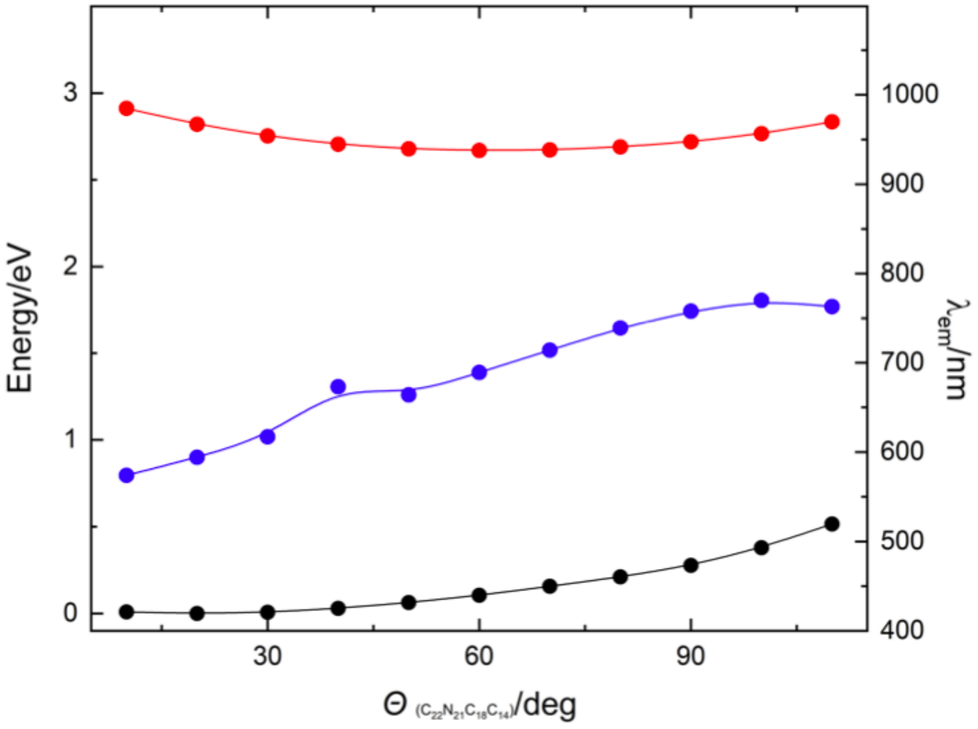

Figure S28: Effect of the dihedral angle $\Theta C_{22}N_{21}C_{18}C_{14}$ on the energy of S_0 (black) and S_1 (red) states of **Py-1H$^+$** in water together with the $S_1 \rightarrow S_0$ emission wavelength (λ_{em}, blue) calculated by the B3LYP/6-31+G(d,p) model.

Table S7: Absorption and emission wavelengths (λ), oscillator strength (f) and molecular orbitals of **Py-2** in water calculated by the CAM-B3LYP/6-31+G(d,p)//B3LYP/6-31+G(d,p) model, together with the experimental absorption maxima.

Transition	λ_{th}/nm	f	MO	%	λ_{exp}/nm
$S_0 \rightarrow T_1$	490	0.0000	$\pi_H \rightarrow \pi_L^*$	59	
$S_0 \rightarrow T_2$	393	0.0000	$\pi_H \rightarrow \pi_{L+2}^*$<br>$\pi_H \rightarrow \pi_{L+3}^*$<br>$\pi_H \rightarrow \pi_{L+5}^*$	14<br>16<br>13	
$S_0 \rightarrow T_3$	371	0.0000	$\pi_{H-4} \rightarrow \pi_{L+6}^*$<br>$\pi_H \rightarrow \pi_{L+8}^*$	11<br>14	
$S_0 \rightarrow S_1$	351	1.2262	$\pi_H \rightarrow \pi_L^*$	86	360
$S_0 \rightarrow T_4$	351	0.0000	$\pi_H \rightarrow \pi_{L+1}^*$	42	
$S_0 \rightarrow T_5$	334	0.0000	$\pi_H \rightarrow \pi_{L+2}^*$	15	
$S_0 \rightarrow T_6$	321	0.0000	$\pi_H \rightarrow \pi_{L+6}^*$	22	
$S_0 \rightarrow T_7$	319	0.0000	$\pi_H \rightarrow \pi_{L+6}^*$	34	
$S_0 \rightarrow S_2$	311	0.0722	$\pi_H \rightarrow \pi_{L+1}^*$	50	
$S_0 \rightarrow T_8$	303	0.0000	$\pi_H \rightarrow \pi_{L+9}^*$	29	
$S_0 \rightarrow T_9$	296	0.0000	$\pi_{H-3} \rightarrow \pi_{L+9}^*$	19	
$S_0 \rightarrow T_{10}$	281	0.0000	$\pi_{H-4} \rightarrow \pi_{L+2}^*$	25	
$S_0 \rightarrow S_3$	280	0.1434	$\pi_H \rightarrow \pi_{L+2}^*$	36	
$S_0 \rightarrow S_4$	275	0.3455	$\pi_H \rightarrow \pi_{L+3}^*$<br>$\pi_H \rightarrow \pi_{L+5}^*$	39<br>37	
$S_0 \rightarrow S_5$	271	0.0449	$\pi_H \rightarrow \pi_{L+6}^*$	76	
$S_0 \rightarrow S_6$	260	0.0649	$\pi_H \rightarrow \pi_{L+9}^*$	34	
$S_0 \rightarrow S_7$	249	0.0567	$\pi_{H-1} \rightarrow \pi_L^*$	20	
$S_0 \rightarrow S_8$	245	0.0134	$\pi_H \rightarrow \pi_{L+3}^*$<br>$\pi_H \rightarrow \pi_{L+5}^*$	26<br>28	
$S_0 \rightarrow S_9$	237	0.0140	$n_{H-7} \rightarrow \pi_L^*$	64	
$S_0 \rightarrow S_{10}$	235	0.0385	$\pi_{H-1} \rightarrow \pi_L^*$<br>$\pi_H \rightarrow \pi_{L+8}^*$	23<br>22	
$S_1 \rightarrow S_0$	577	0.2853	$\pi_H \rightarrow \pi_L^*$	99	578

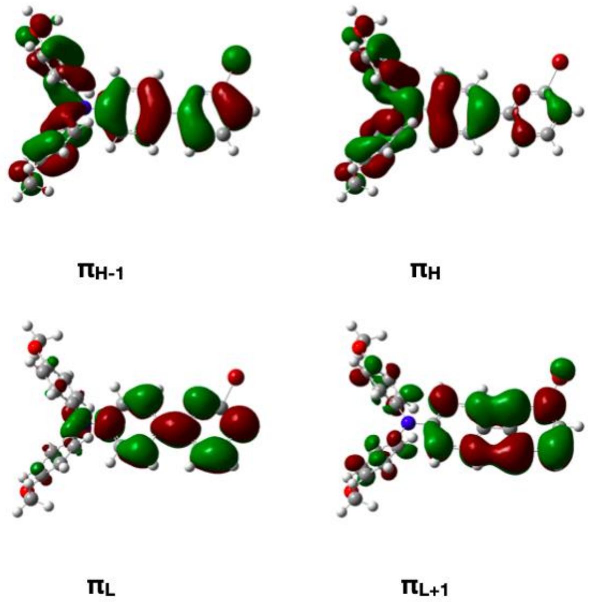

Figure S29: Frontier molecular orbitals of **Py-2** at minS_0 geometry.

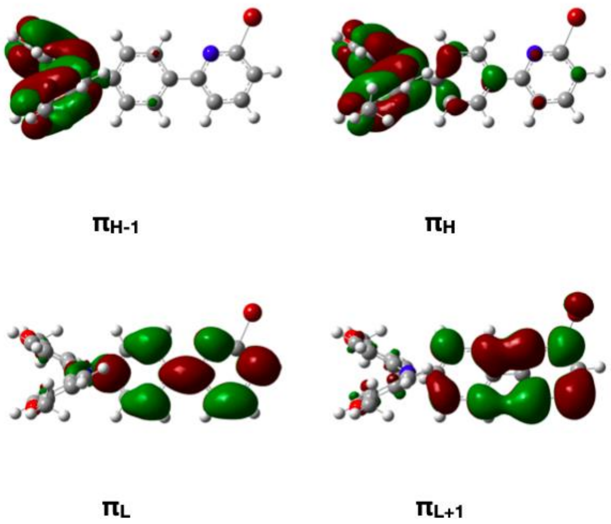

Figure S30: Frontier molecular orbitals of **Py-2** at minS_1 geometry.

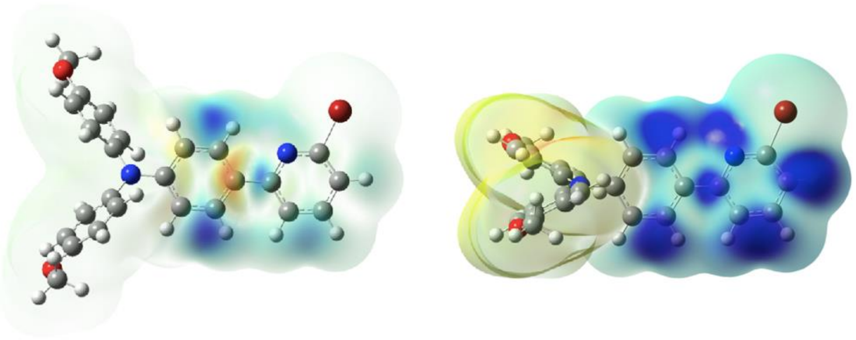

Figure S31: Effect of the $S_0 \rightarrow S_1$ transition on the electron density of **Py-2** at minS_0 (left) and minS_1 (right) geometries; increase and decrease of electron densities are represented by blue (+0.0001) and red (-0.0001), respectively.

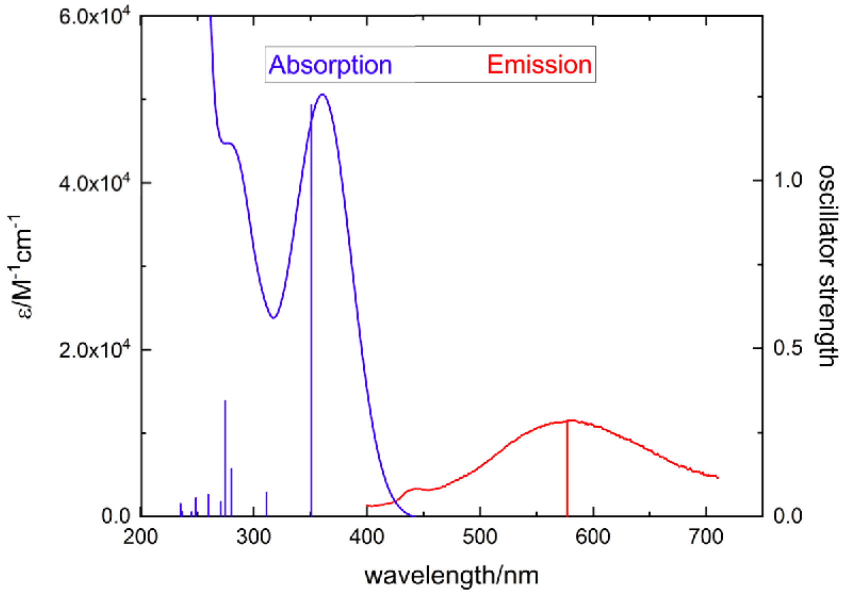

Figure S32: Absorption and emission spectra of **Py-2** (full lines) compared with those calculated by the CAM-B3LYP/6-31+G(d,p)//B3LYP/6-31+G(d,p) model (vertical bars) in water (CPCM).

Table S8: Absorption and emission wavelengths (λ), oscillator strength (f) and molecular orbitals of **Py-2H⁺** in water calculated by the CAM-B3LYP/6-31+G(d,p)//B3LYP/6-31+G(d,p) model, together with the experimental absorption maxima.

Transition	λ_{th}/nm	f	MO	%	λ_{exp}/nm
$S_0 \rightarrow T_1$	545	0.0000	$\pi_H \rightarrow \pi_L^*$	63	
$S_0 \rightarrow S_1$	418	1.1689	$\pi_H \rightarrow \pi_L^*$	81	420
$S_0 \rightarrow T_2$	397	0.0000	$\pi_H \rightarrow \pi_{L+1}^*$	36	
$S_0 \rightarrow T_3$	380	0.0000	$\pi_H \rightarrow \pi_{L+6}^*$	15	
$S_0 \rightarrow T_4$	367	0.0000	$\pi_H \rightarrow \pi_{L+8}^*$	14	
$S_0 \rightarrow T_5$	332	0.0000	$\pi_H \rightarrow \pi_{L+2}^*$	44	
$S_0 \rightarrow T_6$	324	0.0000	$\pi_{H-6} \rightarrow \pi_L^*$	27	
$S_0 \rightarrow T_7$	309	0.0000	$\pi_H \rightarrow \pi_{L+5}^*$	12	
$S_0 \rightarrow S_2$	308	0.1428	$\pi_H \rightarrow \pi_{L+1}^*$	69	
$S_0 \rightarrow T_8$	308	0.0000	$\pi_H \rightarrow \pi_{L+5}^*$	29	
$S_0 \rightarrow T_9$	305	0.0000	$\pi_H \rightarrow \pi_{L+7}^*$	30	
$S_0 \rightarrow T_{10}$	295	0.0000	$\pi_{H-5} \rightarrow \pi_L^*$	53	
$S_0 \rightarrow S_3$	289	0.0795	$\pi_H \rightarrow \pi_{L+2}^*$	63	
$S_0 \rightarrow S_4$	277	0.1121	$\pi_{H-2} \rightarrow \pi_L^*$	56	
$S_0 \rightarrow S_5$	265	0.1775	$\pi_{H-5} \rightarrow \pi_L^*$	29	
$S_0 \rightarrow S_6$	261	0.0299	$\pi_H \rightarrow \pi_{L+5}^*$	56	
$S_0 \rightarrow S_7$	257	0.3073	$\pi_H \rightarrow \pi_{L+6}^*$<br>$\pi_H \rightarrow \pi_{L+7}^*$	23<br>22	
$S_0 \rightarrow S_8$	251	0.1071	$\pi_{H-1} \rightarrow \pi_L^*$	46	
$S_0 \rightarrow S_9$	247	0.1756	$\pi_{H-5} \rightarrow \pi_L^*$<br>$\pi_{H-1} \rightarrow \pi_L^*$	23<br>33	
$S_0 \rightarrow S_{10}$	242	0.0679	$\pi_H \rightarrow \pi_{L+8}^*$	58	

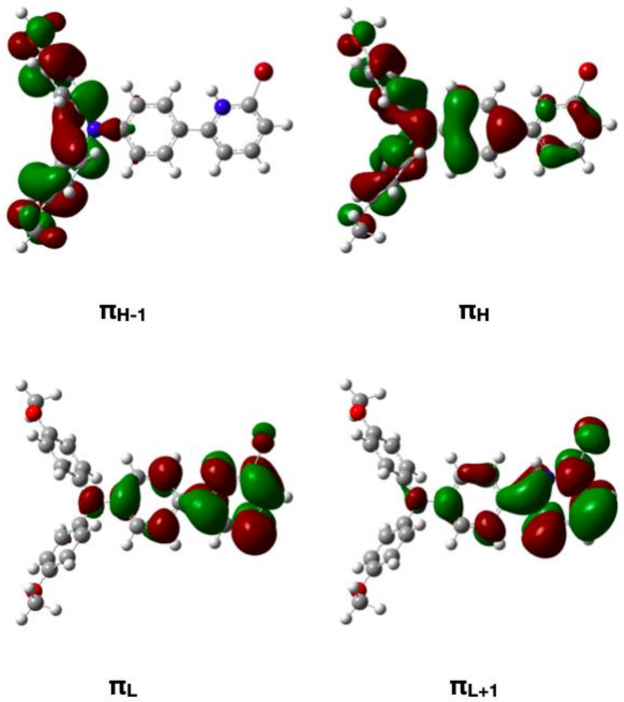

Figure S33: Frontier molecular orbitals of **Py-2H⁺** at minS_0 geometry.

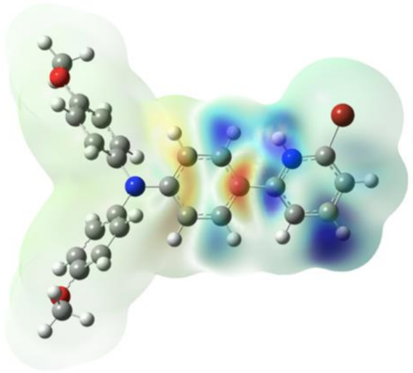

Figure S34: Effect of the $S_0 \rightarrow S_1$ transition on the electron density of **Py-2H⁺** at minS_0 geometry; increase and decrease of electron densities are represented by blue (+0.0001) and red (-0.0001), respectively.

Table S9: Absorption and emission wavelengths (λ), oscillator strength (f) and molecular orbitals of **Py-3** in water calculated by the CAM-B3LYP/6-31+G(d,p)//B3LYP/6-31+G(d,p) model, together with the experimental absorption maxima.

Transition	λ_{th}/nm	f	MO	%	λ_{exp}/nm
$S_0 \rightarrow T_1$	478	0.0000	$\pi_H \rightarrow \pi_L^*$	39	
$S_0 \rightarrow T_2$	443	0.0000	$\pi_{H-1} \rightarrow \pi_L^*$<br>$\pi_H \rightarrow \pi_{L+1}^*$	25<br>32	
$S_0 \rightarrow T_3$	397	0.0000	$\pi_H \rightarrow \pi_{L+5}^*$	13	
$S_0 \rightarrow T_4$	397	0.0000	$\pi_{H-1} \rightarrow \pi_{L+5}^*$	14	
$S_0 \rightarrow T_5$	374	0.0000	$\pi_{H-2} \rightarrow \pi_L^*$	10	
$S_0 \rightarrow T_6$	365	0.0000	$\pi_{H-3} \rightarrow \pi_{L+5}^*$	8	
$S_0 \rightarrow T_7$	354	0.0000	$\pi_{H-1} \rightarrow \pi_{L+2}^*$<br>$\pi_H \rightarrow \pi_{L+3}^*$	29<br>30	
$S_0 \rightarrow T_8$	354	0.0000	$\pi_{H-1} \rightarrow \pi_{L+3}^*$<br>$\pi_H \rightarrow \pi_{L+2}^*$	28<br>31	
$S_0 \rightarrow S_1$	338	1.5254	$\pi_H \rightarrow \pi_{L+1}^*$	52	360
$S_0 \rightarrow T_9$	333	0.0000	$\pi_{H-13} \rightarrow \pi_L^*$<br>$\pi_{H-1} \rightarrow \pi_{L+1}^*$	10<br>10	
$S_0 \rightarrow S_2$	330	0.6175	$\pi_H \rightarrow \pi_L^*$	58	
$S_0 \rightarrow T_{10}$	324	0.0000	$\pi_H \rightarrow \pi_{L+9}^*$	18	
$S_0 \rightarrow S_3$	315	0.1708	$\pi_{H-1} \rightarrow \pi_{L+2}^*$<br>$\pi_H \rightarrow \pi_{L+3}^*$	39<br>44	
$S_0 \rightarrow S_4$	314	0.0457	$\pi_{H-1} \rightarrow \pi_{L+3}^*$<br>$\pi_H \rightarrow \pi_{L+2}^*$	39<br>45	
$S_0 \rightarrow S_5$	285	0.0717	$\pi_{H-1} \rightarrow \pi_L^*$	33	
$S_0 \rightarrow S_6$	280	0.2480	$\pi_{H-1} \rightarrow \pi_{L+5}^*$	42	
$S_0 \rightarrow S_7$	280	0.7077	$\pi_H \rightarrow \pi_{L+5}^*$	43	
$S_0 \rightarrow S_8$	274	0.0064	$\pi_{H-1} \rightarrow \pi_{L+1}^*$	26	
$S_0 \rightarrow S_9$	273	0.0845	$\pi_{H-1} \rightarrow \pi_{L+8}^*$<br>$\pi_H \rightarrow \pi_{L+9}^*$	38<br>36	
$S_0 \rightarrow S_{10}$	273	0.0351	$\pi_{H-1} \rightarrow \pi_{L+9}^*$<br>$\pi_H \rightarrow \pi_{L+8}^*$	34<br>38	
$S_1 \rightarrow S_0$	489	0.7341	$\pi_H \rightarrow \pi_L^*$	86	527

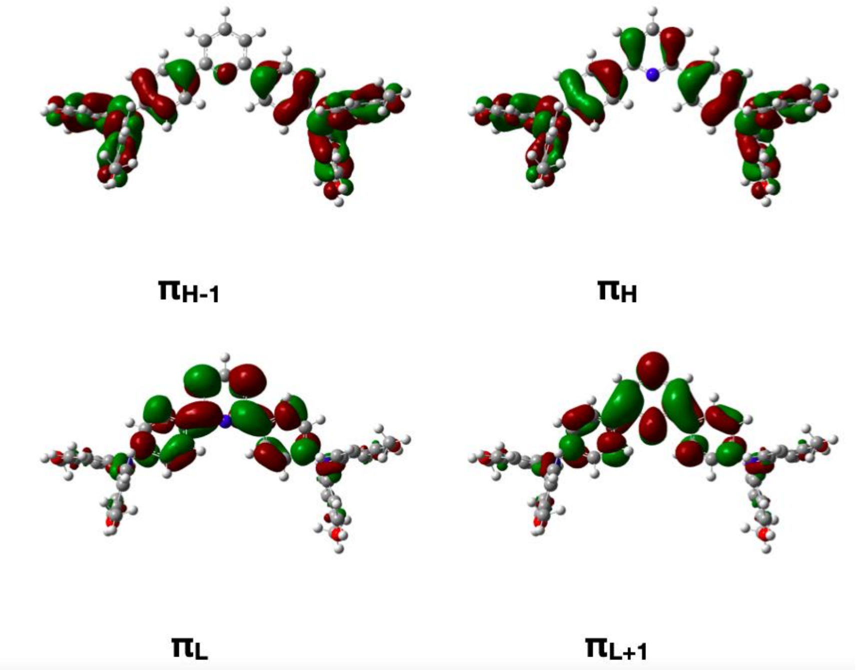

Figure S35: Frontier molecular orbitals of **Py-3** at minS_0 geometry.

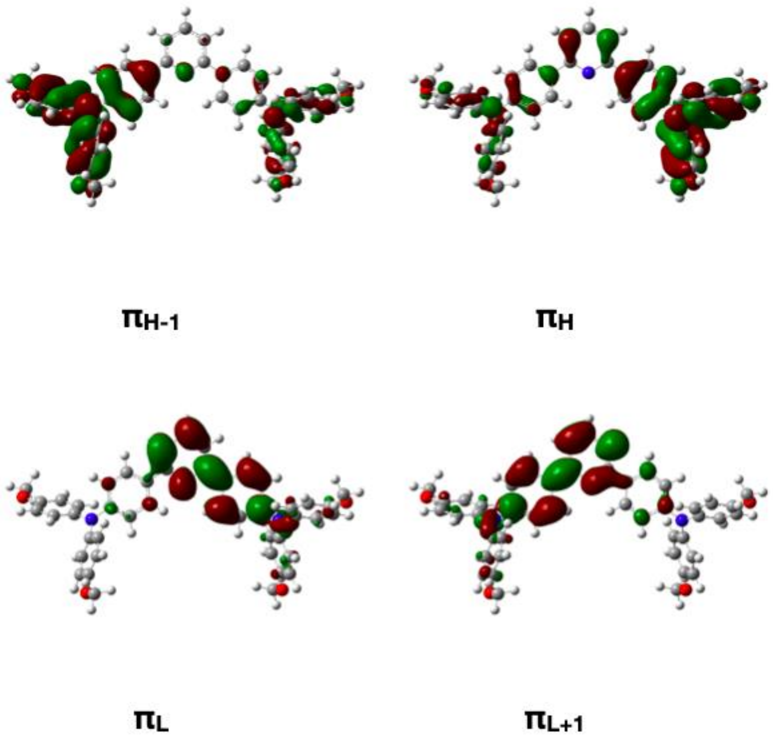

Figure S36: Frontier molecular orbitals of **Py-3** at minS_1 geometry.

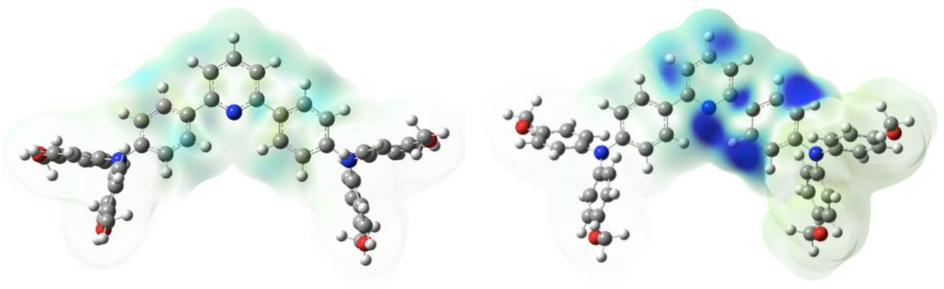

Figure S37: Effect of the $S_0 \rightarrow S_1$ transition on the electron density of **Py-3** at minS_0 geometry; increase and decrease of electron densities are represented by blue (+0.0001) and red (-0.0001), respectively.

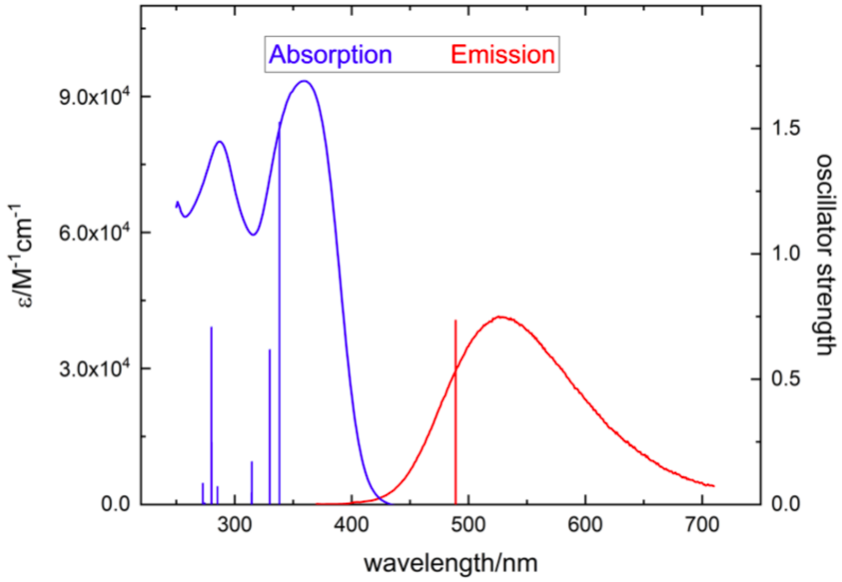

Figure S38: Absorption and emission spectra of **Py-3** (full lines) compared with those calculated by the CAM-B3LYP/6-31+G(d,p)//B3LYP/6-31+G(d,p) model (vertical bars) in water (CPCM).

Table S10: Absorption and emission wavelengths (λ), oscillator strength (f) and molecular orbitals of **Py-3H$^+$** in water calculated by the CAM-B3LYP/6-31+G(d,p)//B3LYP/6-31+G(d,p) model, together with the experimental absorption maxima.

Transition	λ_{th}/nm	f	MO	%	λ_{exp}/nm
$S_0 \to T_1$	512	0.0000	$\pi_H \to \pi_L^*$	56	
$S_0 \to T_2$	496	0.0000	$\pi_H \to \pi_{L+1}^*$	39	
$S_0 \to S_1$	417	1.3526	$\pi_H \to \pi_L^*$	76	443
$S_0 \to T_3$	385	0.0000	$\pi_H \to \pi_{L+6}^*$	12	
$S_0 \to T_4$	385	0.0000	$\pi_{H-1} \to \pi_{L+6}^*$	13	
$S_0 \to T_5$	377	0.0000	$\pi_H \to \pi_{L+5}^*$	14	
$S_0 \to T_6$	372	0.0000	$\pi_{H-1} \to \pi_L^*$	16	
$S_0 \to S_2$	365	0.6416	$\pi_{H-1} \to \pi_L^*$	61	
$S_0 \to T_7$	360	0.0000	$\pi_{H-1} \to \pi_L^*$	28	
$S_0 \to T_8$	351	0.0000	$\pi_{H-1} \to \pi_{L+1}^*$	13	
$S_0 \to T_9$	338	0.0000	$\pi_{H-1} \to \pi_{L+2}^*$<br>$\pi_H \to \pi_{L+3}^*$	19<br>21	
$S_0 \to T_{10}$	338	0.0000	$\pi_H \to \pi_{L+2}^*$	23	
$S_0 \to S_3$	312	0.0740	$\pi_H \to \pi_{L+1}^*$	51	
$S_0 \to S_4$	306	0.2813	$\pi_{H-1} \to \pi_{L+1}^*$	51	
$S_0 \to S_5$	298	0.0631	$\pi_H \to \pi_{L+2}^*$	44	
$S_0 \to S_6$	297	0.0966	$\pi_{H-1} \to \pi_{L+2}^*$<br>$\pi_H \to \pi_{L+3}^*$	31<br>31	
$S_0 \to S_7$	267	0.1074	$\pi_{H-2} \to \pi_L^*$	55	
$S_0 \to S_8$	267	0.1292	$\pi_H \to \pi_{L+7}^*$	29	
$S_0 \to S_9$	266	0.6225	$\pi_H \to \pi_{L+6}^*$	28	
$S_0 \to S_{10}$	264	0.0328	$\pi_{H-1} \to \pi_{L+9}^*$<br>$\pi_H \to \pi_{L+8}^*$	33<br>35	

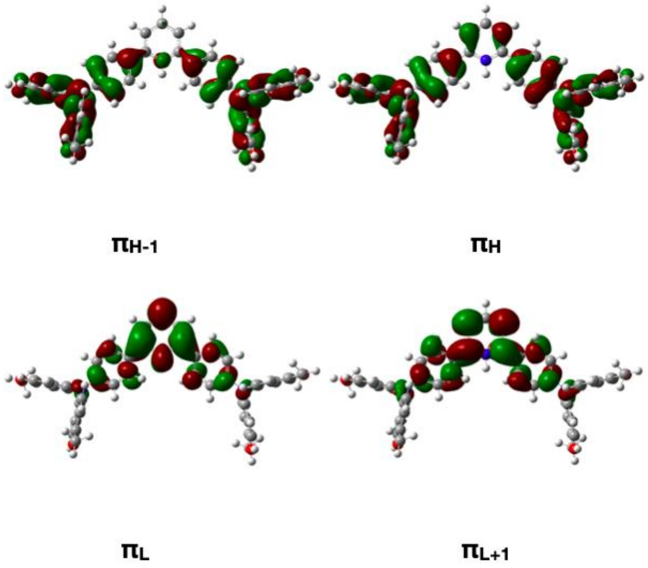

Figure S39: Frontier molecular orbitals of **Py-3H$^+$** at minS$_0$ geometry.

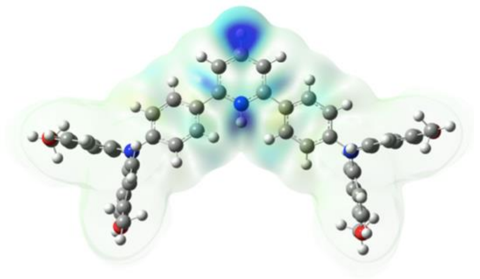

Figure S40: Effect of the S$_0$→S$_1$ transition on the electron density of **Py-3H$^+$** at minS$_0$ geometry; increase and decrease of electron densities are represented by blue (+0.0001) and red (-0.0001), respectively.

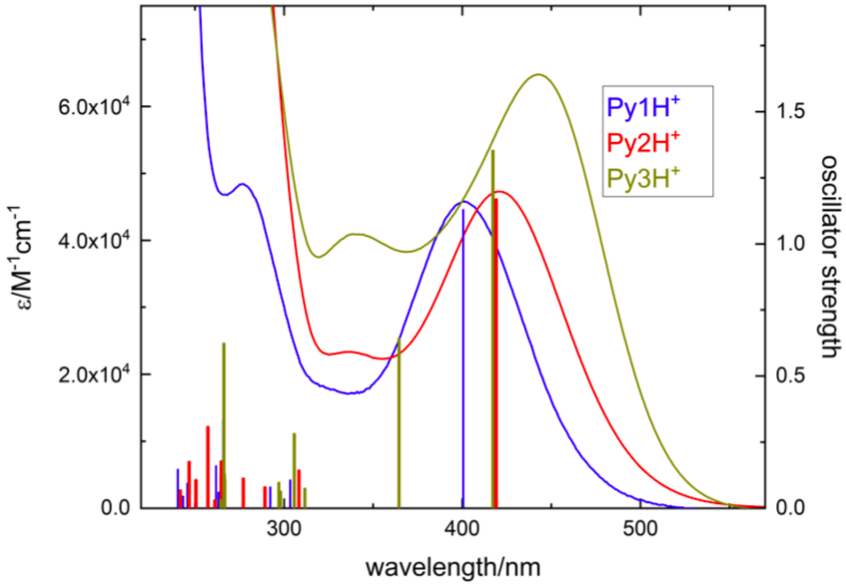

Figure S41: Absorption spectra of **Py-1H$^+$**, **Py-2H$^+$** and **Py-3H$^+$** in water (CPCM) calculated by the CAM- B3LYP/6-31+G(d,p)//B3LYP/6-31+G(d,p) model (full lines and vertical bars) compared with the experimental spectra (dashed lines).

Table S11. Results of the Natural Transition Orbital (NTO) analysis for S_1 on the ground state optimized geometry (minS_0) in terms of occupation numbers.

Compound	Configuration	Occupation Number
**Py-1**	$\pi_H \rightarrow \pi_L^*$	0.96151
**Py-1H$^+$**	$\pi_H \rightarrow \pi_L^*$	0.97792
**Py-2**	$\pi_H \rightarrow \pi_L^*$	0.96401
**Py-2H$^+$**	$\pi_H \rightarrow \pi_L^*$	0.97777
**Py-3**	$\pi_H \rightarrow \pi_L^*$<br>$\pi_{H-1} \rightarrow \pi_{L+1}^*$	0.60755<br>0.35581
**Py-3H$^+$**	$\pi_H \rightarrow \pi_L^*$<br>$\pi_{H-1} \rightarrow \pi_{L+1}^*$	0.89137<br>0.09250
